# A review of potential biomarkers for assessing physical and psychological trauma in paediatric burns

**DOI:** 10.1093/burnst/tkaa049

**Published:** 2021-02-09

**Authors:** Morgan Carlton, Joanne Voisey, Tony J Parker, Chamindie Punyadeera, Leila Cuttle

**Affiliations:** Queensland University of Technology (QUT), Centre for Children’s Burn and Trauma Research, Centre for Children’s Health Research, South Brisbane, Queensland, Australia; Queensland University of Technology (QUT), Faculty of Health, School of Biomedical Sciences, Brisbane, Queensland, Australia; Queensland University of Technology (QUT), Faculty of Health, School of Biomedical Sciences, Brisbane, Queensland, Australia; Queensland University of Technology (QUT), Faculty of Health, School of Biomedical Sciences, Brisbane, Queensland, Australia; Queensland University of Technology (QUT), Faculty of Health, School of Biomedical Sciences, Saliva and Liquid Biopsy Translational Laboratory, Brisbane, Queensland, Australia; Queensland University of Technology (QUT), Centre for Children’s Burn and Trauma Research, Centre for Children’s Health Research, South Brisbane, Queensland, Australia; Queensland University of Technology (QUT), Faculty of Health, School of Biomedical Sciences, Brisbane, Queensland, Australia

**Keywords:** Paediatric burns, Biomarker, Inflammatory, Hypothalamic-pituitary-adrenal axis, Physical trauma, Psychological trauma

## Abstract

Biological markers that evaluate physical healing as well as psychological impact of a burn are essential for effective treatment of paediatric burns. The objective of this review is to summarize the evidence supporting the use of biomarkers in children with burns. An extensive review of the literature was performed using PubMed. A total of 59 biomarkers were identified relating to burn presence, specifically relating to processes involved in inflammation, wound healing, growth and metabolism. In addition, biomarkers involved in the stress response cascade following a burn trauma were also identified. Although many biomarkers have been identified that are potentially associated with burn-related physical and psychological trauma, an understanding of burn biology is still lacking in children. We propose that future research in the field of children’s burns should be conducted using broad screening methods for identifying potential biomarkers, examine the biological interactions of different biomarkers, utilize child-appropriate biological fluids such as urine or saliva, and include a range of different severity burns. Through further research, the biological response to burn injury may be fully realized and clinically relevant diagnostic tests and treatment therapies utilizing these biomarkers could be developed, for the improvement of healing outcomes in paediatric burn patients.

Highlights

- Over 70 potential biomarkers have been investigated in paediatric burns.
- Inflammation, metabolism and stress responses are heightened following a burn.
- Few markers have been evaluated in child-friendly, non-invasive biological mediums.

## Background

Burn injuries are devastating for children, due to the extensive treatment requirements and the life-long complications that accompany them. Treatment includes extremely painful wound debridement, numerous dressings and in more severe cases, grafting procedures are also required. For many paediatric patients, the initial healing stage is followed by years of scar management and reconstructive surgeries to prevent complications in physical development [1] and reduce the burn’s severe impact on quality of life [2].

Burn injuries have a dual impact on the injured individual. Most obviously is the effect of the injury on the physiology of the body, both local and systemic [3, 4]. Not so obvious are the effects on the mental well-being of the patient, due to pain, stress and anxiety. Evidence shows that psychological distress is associated with delayed physical healing [5, 6]; however, more importantly, burns can increase the risk of an individual later developing mental health issues such as post-traumatic stress disorder (PTSD) [7–9]. It is important to identify individuals who are at risk of such disorders early to provide treatment and implement preventative approaches.

Burn wound healing is often assessed by clinicians observing the physical appearance of the wound site. This is a subjective measure that relies on the experience of the attending physician. Similarly, the assessment of a patient’s stress and anxiety levels relies heavily on patient self-reporting through questionnaires, or observations from family members and nurses using pain scales for non-vocal children under the age of two [8, 10–14]. These reports are also subjective and can introduce bias. Unfortunately, there are few documented or validated objective tools available to replace these assessment methods. The identification and measurement of biomarkers present in biological fluids have the potential to allow clinicians to diagnose and monitor the healing progression of children with burns accurately and objectively. However, there are currently no commercially available diagnostic and prognostic tests for use in the clinic. This review documents the biomarkers that have been investigated in paediatric burns and comments on the future of paediatric burn biomarker utilization.

### Paediatric burn biomarker research

A biomarker is defined as a chemical, its metabolite, or the product of an interaction between a chemical and some target molecule or cell that is measured in the human body [15]. Biomarkers can provide information that may be indicative of normal biological processes, disease states or responses to therapeutic interventions [16]. Consequently, by utilizing knowledge of the biological pathways underpinning burn injuries, biomarkers may be identified that could objectively classify burn severity, predict healing trajectory, and monitor healing progression. Furthermore, they could be used to identify susceptibility to comorbidities such as sepsis or PTSD. Already, specific biomarkers are being investigated in paediatric burns to achieve some of these outcomes [17, 18].

**Figure 1.**
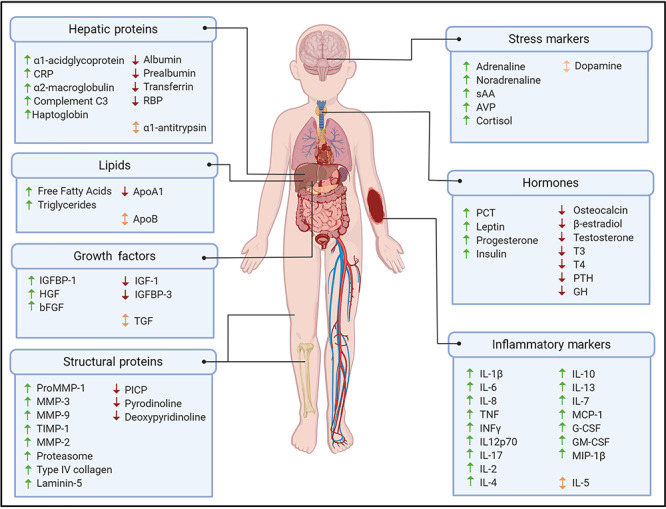
Summary of reported systemic biomarker changes in response to paediatric burn injury. Up arrows (↑) indicate increased abundance of biomarker following a burn in children; down arrows (↓) indicate reduced abundance of biomarker following a burn in children; and bidirectional arrows (↕) indicate conflicting evidence for biomarker abundance following a burn in children. Image created with BioRender.com. *CRP* C-reactive protein, *RBP* retinol binding protein, *sAA* salivary alpha-amylase, *AVP* arginine vasopressin, *IGF* insulin-like growth factor, *IGFBP* insulin-like growth factor binding protein, *HGF* hepatocyte growth factor, *bFGF* basic fibroblast growth factor, *TGF* transforming growth factor, *PCT* procalcitonin, *T3* triiodothyronine, *T4* thyroxine, *PTH* parathyroid hormone, *GH* growth hormone, *MMP* matrix metalloproteases, *PICP* carboxyterminal propeptide of type I procollagen, *TIMP-1* tissue inhibitor of metalloproteinases-1, *IL* interleukin, *TNF* tumour necrosis factor, *INFγ* interferon-gamma, *MCP-1* monocyte chemoattractant protein-1, *G-CSF* granulocyte-colony stimulating factor, *GM-CSF* granulocyte-macrophage colony-stimulating factor, *MIP-1β* macrophage inflammatory protein 1β

Over the past 35 years, numerous biomarkers have been investigated in paediatric burns (Figure 1) with many evaluated by comparing the biomarker abundance in children with burns to that in healthy children without burns. Some markers have been quantified at multiple time points across the healing process and compared to ‘normal’ ranges while others have been investigated in terms of their relationship to specific variables, such as burn severity, sepsis, survival or stress. Biomarkers have primarily been investigated in blood [19–22]; however, other biological fluids such as urine [23, 24], blister fluid [25, 26] and saliva [7] have also been evaluated. Numerous markers are being analysed to determine how they are affected by burn injury and how they may relate to burn outcome; however, understanding the role that each marker plays in thermal injury response is complex. Rarely does a marker have one specific role, instead, they often participate in many different physiological processes. To simplify the information presented in this review, the markers have been categorized into potential biomarkers for evaluating inflammation, tissue repair/wound healing, growth and metabolism, and stress.

### Methodology

A review of the literature was performed to identify the biomarkers that have been investigated in children’s burns. A PubMed search was conducted using the terms (burn OR ‘thermal injury’ OR scald), (paediatric OR pediatric OR child OR children OR youth OR adolescent), (biomarker OR marker), (saliva OR blood OR plasma OR serum OR hair OR urine OR eschar OR ‘blister fluid’ OR ‘cerebrospinal fluid’), (human), (stress OR pain OR distress OR psycolog^*^ OR anxiety), (sepsis), (severity OR healing OR re-epithelialization OR reepithelialisation) and (survival OR mortality). The initial search returned 410 studies. The returned studies were screened for relevance and were excluded if they were: performed in adults, animals or cell lines; not focused on biomarker investigation; evaluated the effects of treatment; or written in languages other than English. The remaining studies were reviewed, and additional relevant studies were identified through manually searching the reference lists of the reviewed articles and added to the review (n = 41).

As burn mortality rates have continued to improve, research has focused more on investigating the biological response to burns, including the identification of markers related to clinical outcomes, such as sepsis, scarring and long-term co-morbidities [27]. As such, the focus of this review is to discuss biomarkers that have been associated with burn injury to better understand the underlying biological impacts of burns in children. Biomarkers specifically implicated in patient mortality or burn-related septic events and other co-morbidities are not reviewed in detail, as these biomarkers are discussed elsewhere [28–30].

## Review

### Biomarkers for evaluating inflammation

It is well known that burn injury initiates a systemic inflammatory response that subsequently alters many essential homeostatic processes. Leaving the inflammatory response unchecked can result in increased susceptibility to infection, multiple organ failure and death. Therefore, there is a crucial need to understand the post-burn inflammatory response, how it affects other bodily systems and specifically what markers are involved, to develop therapies that mitigate these outcomes.

Numerous markers associated with the inflammatory response have been investigated in paediatric burns to better understand the post-burn inflammatory response in children (Table 1). After burn injury, inflammatory cytokines involved in both acute phase, such as interleukin (IL)-1, IL-6, tumour necrosis factor (TNF) and interferon gamma (IFNγ); and chronic inflammation, such as IL-2, IL-3, IL-5, IL-7, IL-10, IL12, IL-13 and transforming growth factor (TGF); are increased [31, 32]. Expression of both pro-inflammatory and anti-inflammatory cytokines is altered immediately following the burn, is sustained for several months and affects several other physiological processes. Inflammatory pathways are triggered after a physical injury such as a burn but when children experience psychological trauma inflammation also occurs. A recent publication in *Nature Medicine* highlights that chronic inflammation traced back to early development can lead to numerous mental and physical health problems [33].

**Table 1:** Summary of reported abundance of biomarkers involved in the inflammatory response in children with burns compared to healthy children without burns

**Reference**	**Source**	**Reported normal limits**	**Abundance in children with burns**	**Age range**	**Time frame**	**Population TBSA (%)**
**IL-17**						
Jeschke *et al.* (2008a) [37]	Blood	Undetectable^†^	Elevated 0.6–2.75 ng/mL^†^	8.0 ± 0.2 years	Up to 60 days post-burn	56 ± 0.3^a^
Finnerty *et al.* (2006) [36]	Blood	0.1 ± 0.0 pg/mL	Elevated 17 pg/mL^†^	2–15 years	Immediately after burn	50 ± 3^a^
Jeschke *et al.* (2011) [38]	Blood	<1 pg/mL^†^	Elevated 4.1–9.5 pg/mL^†^	7.5 ± 5.3 years	Up to 1100 days post-burn	50 ± 20^b^
**IL-1β**						
Jeschke *et al.* (2008a) [37]	Blood	0.9 ng/mL^†^	Elevated 2.4 ng/mL^†^	8.0 ± 0.2 years	Immediately after burn	56 ± 0.3^a^
Finnerty *et al.* (2006) [36]	Blood	0.9 ± 0.1 pg/mL	Elevated 7 pg/mL^†^	2–15 years	Immediately after burn	50 ± 3^a^
Klein *et al.* (1995) [41]	Blood	<1 pg/mL	Elevated 3.4 ± 1.9 pg/mL	5.8–17.5 years	3 weeks post-burn	63 ± 16^a^
Jeschke *et al.* (2004) [40]	Blood	Not reported	Elevated 1.75–2.75 pg/mL^†^	5.7 ± 3.9 years	Up to 40 days post-burn	67 ± 14^b^
Jeschke *et al.* (2011) [38]	Blood	2 pg/mL^†^	Elevated 5–20 pg/mL^†^	7.5 ± 5.3 years	Up to 60 days post-burn	50 ± 20^b^
**TNF**						
Jeschke *et al.* (2008a) [37]	Blood	0.7 ng/mL^†^	Elevated 2.5–3.5 ng/mL^†^	8.0 ± 0.2 years	Up to 7 days post-burn	56 ± 0.3^a^
Finnerty *et al.* (2006) [36]	Blood	0.5 pg/mL	Within normal limits 0.25–4.5 pg/mL	2–15 years	Within first 4 weeks post-burn	50 ± 3^a^
Jeschke *et al.* (2004) [40]	Blood	Not reported	Elevated 3–13 pg/mL	5.7 ± 3.9 years	Up to 40 days post-burn	67 ± 14^b^
Kulp *et al.* (2010) [24]	Urine (in 24 hours)	5 pg/mL^†^	Elevated 14–25 pg/mL^†^	8 ± 5 years	Up to 180 days post-burn	59 ± 17^a^
Abdel-Hafez *et al.* (2007) [20]	Blood	7.74 ± 3.03 ng/L	Elevated 98.3 ± 15.4 ng/L	2 months-7 years	At admission	31.62 ± 12.01^b^
Jeschke *et al.* (2011) [38]	Blood	8 pg/mL^†^	Elevated 17.5–38 pg/mL^†^	7.5 ± 5.3 years	From admission to 16 days post-burn, then fluctuates up to 1100 days post-burn	50 ± 20^b^
**IL-6**						
Jeschke *et al.* (2008a) [37]	Blood	<10 ng/mL^†^	Elevated 380–1150 ng/mL^†^	8.0 ± 0.2 years	Up to 60 days post-burn	56 ± 0.3^a^
Finnerty *et al.* (2006) [36]	Blood	4.1 ± 1.7 pg/mL	Elevated 300–1800 pg/mL^†^	2–15 years	Up to 4 weeks post-burn	50 ± 3^a^
Klein *et al.* (1995) [41]	Blood	<1 pg/mL	Elevated 126 ± 58 pg/mL	5.8–17.5 years	3 weeks post-burn	63 ± 16^a^
Jeschke *et al.* (2012a) [45]	Blood	<10 ng/mL^†^	Elevated 280–1020 ng/mL^†^	8 ± 5 years	Up to 250 days post-burn	64 ± 12^b^
Jeschke *et al.* (2004) [40]	Blood	Not reported	Elevated 60–80 pg/mL^†^	5.7 ± 3.9 years	Up to 10 days post-burn	67 ± 14^b^
Kulp *et al.* (2010) [24]	Urine (in 24 hours)	<50 pg/mL^†^	Elevated 100–3000 pg/mL^†^	8 ± 5 years	Up to 180 days post-burn	59 ± 17^a^
Gauglitz *et al.* (2009) [43]	Blood	<50 pg/mL^†^	Elevated 1100–2200 pg/mL^†^	8.8 ± 5.3 years	Up to 2 months post-burn	57.9 ± 14.7^b^
Abdel-Hafez *et al.* (2007) [20]	Blood	12.4 ± 5.7 pg/mL	Elevated 145.3 ± 36.4 pg/mL	2 months-7 years	At admission	31.62 ± 12.01^b^
Jeschke *et al.* (2011) [38]	Blood	Undetectable^†^	Elevated 50–2650 pg/mL^†^	7.5 ± 5.3 years	Up to 1100 days post-burn	50 ± 20^b^
**α1-Acid glycoprotein**						
Jeschke *et al.* (2004) [40]	Blood	Not stated	Elevated 200–255 mg/dl^†^	1–16 years	From 5 to 80 days post-burn	67 ± 14^b^
Jeschke *et al.* (2008a) [37]	Blood	60 ng/mL^†^	Elevated 125–225 ng/mL^†^	8.0 ± 0.2 years	Up to 60 days post-burn	56 ± 0.3^a^
Klein *et al.* (1995) [41]	Blood	0.55 ± 1.40 g/L	Elevated 2.00 ± 0.34 g/L	5.8–17.5 years	3 weeks post-burn	63 ± 16^a^
Jeschke *et al.* (2011) [38]	Blood	100 mg/dL^†^	Elevated 200–220 mg/dL^†^	7.5 ± 5.3 years	From 8 days to 90 days post-burn	50 ± 20^b^
**C-reactive protein**						
Jeschke *et al.* (2008a) [37]	Blood	<1 ng/mL^†^	Elevated 9–14.5 ng/mL^†^	8.0 ± 0.2 years	Up to 60 days post-burn	56 ± 0.3^a^
Jeschke *et al.* (2004) [40]	Blood	Not Stated	Elevated 7–17 mg/dl^†^	5.7 ± 3.9 years	Up to 70 days post-burn	67 ± 14^b^
Abdel-Hafez *et al.* (2007) [20]	Blood	2.4 ± 0.40 μg/mL	Elevated 32.12 ± 19.08 μg/mL	2 months-7 years	At admission	31.62 ± 12.01^b^
Jeschke *et al.* (2011) [38]	Blood	<0.5 mg/dL^†^	Elevated 1.5–13.5 mg/dL^†^	7.5 ± 5.3 years	Up to 270 days post-burn	50 ± 20^b^
**α2-Macroglobulin**						
Jeschke *et al.* (2008a) [37]	Blood	150 ng/mL	Elevated 175 ng/mL	8.0 ± 0.2 years	At day 35–60 post-burn	56 ± 0.3^a^
Jeschke *et al.* (2011) [38]	Blood	267.5 mg/dL^†^	Reduced 120–180 mg/dL^†^	7.5 ± 5.3 years	Up to 60 days post-burn	50 ± 20^b^
**Complement C3**						
Jeschke *et al.* (2008a) [37]	Blood	130 ng/mL^†^	Elevated 150–170 ng/mL^†^	8.0 ± 0.2 years	17–60 days post-burn	56 ± 0.3^a^
Jeschke *et al.* (2011) [38]	Blood	140 mg/dL^†^	Reduced 90–120 mg/dL^†^	7.5 ± 5.3 years	From admission to 10 days post-burn	50 ± 20^b^
Jeschke *et al.* (2011) [38]	Blood	140 mg/dL^†^	Elevated 165–185 mg/dL^†^	7.5 ± 5.3 years	Days 29–90 post-burn	50 ± 20^b^
**α1-Antitrypsin**						
Jeschke *et al.* (2004) [40]	Blood	Not stated	Elevated 280–370 mg/dl^†^	1–16 years	From 5 to 80 days post-burn	67 ± 14^b^
Klein *et al.* (1995) [41]	Blood	1.9 ± 3.5 g/L	Elevated 3.69 ± 1.01 g/L	5.8–17.5 years	3 weeks post-burn	63 ± 16^a^
**Haptoglobin**						
Jeschke *et al.* (2004) [40]	Blood	Not stated	Elevated 280–475 mg/dl^†^	1–16 years	From 5 to 80 days post-burn	67 ± 14^b^
Jeschke *et al.* (2008a) [37]	Blood	105 ng/mL^†^	Elevated 245–370 ng/mL^†^	8.0 ± 0.2 years	From 2 to 7 days post-burn up to 60 days post-burn	56 ± 0.3^a^
Jeschke *et al.* (2011) [38]	Blood	160 mg/dL^†^	Elevated 280–335 mg/dL^†^	7.5 ± 5.3 years	From 8 days to 90 days post-burn	50 ± 20^b^
**Leptin**						
Abdel-Hafez *et al.* (2007) [20]	Blood	1.3 ± 0.4 ng/mL	Elevated 15.7 ± 1.28 ng/mL	2 months-7 years	At admission	31.62 ± 12.01^b^
**IFN γ**						
Jeschke *et al.* (2008a) [37]	Blood	2 ng/mL^†^	Elevated 5–16 ng/mL^†^	8.0 ± 0.2 years	Until 7 days post-burn, at 11–16 days post-burn, and at 23–28 days post-burn.	56 ± 0.3^a^
Finnerty *et al.* (2006) [36]	Blood	1.5 ± 0.5 pg/mL	Elevated 52 pg/mL^†^	2–15 years	Immediately after burn	50 ± 3^a^
Jeschke *et al.* (2011) [38]	Blood	5 pg/mL^†^	Elevated 22.5–67.5 pg/mL^†^	7.5 ± 5.3 years	Up to 1100 days post-burn	50 ± 20^b^
**IL-12p70**						
Jeschke *et al.* (2008a) [37]	Blood	Undetectable^†^	Elevated 0.35–1.4 ng/mL^†^	8.0 ± 0.2 years	Up to 60 days post-burn	56 ± 0.3^a^
Finnerty *et al.* (2006) [36]	Blood	Undetectable	Elevated 2–2.3 pg/mL^†^	2–15 years	Immediately after burn and at 3 weeks post-burn	50 ± 3^a^
Jeschke *et al.* (2011) [38]	Blood	7.5 pg/mL^†^	Within normal limits	7.5 ± 5.3 years	Up to 1100 days post-burn	50 ± 20^b^
**Procalcitonin**						
Abdel-Hafez *et al.* (2007) [20]	Blood	0.17 ± 0.02 ng/mL	Elevated 69.1 ± 11.4 ng/mL	2 months-7 years	At admission	31.62 ± 12.01^b^
**MCP-1**						
Jeschke *et al.* (2008a) [37]	Blood	40 ng/mL^†^	Elevated 110–200 ng/mL^†^	8.0 ± 0.2 years	Up to 60 days post-burn	56 ± 0.3^a^
Finnerty *et al.* (2006) [36]	Blood	41.9 ± 5.4 pg/mL	Elevated 140–280 pg/mL^†^	2–15 years	Up to 1-week post-burn	50 ± 3^a^
Gauglitz *et al.* (2009) [43]	Blood	50 pg/mL^†^	Elevated 70–350 pg/mL^†^	8.8 ± 5.3 years	Up to 36 months post-burn	57.9 ± 14.7^b^
Jeschke *et al.* (2012a) [45]	Blood	50 ng/mL^†^	Elevated 75–640 ng/mL	8 ± 5 years	Up to 250 days post-burn	64 ± 12^b^
Jeschke *et al.* (2011) [38]	Blood	80 pg/mL^†^	Elevated 125–550 pg/mL^†^	7.5 ± 5.3 years	Up to 1100 days post-burn	50 ± 20^b^
**MIP-1β**						
Jeschke *et al.* (2008a) [37]	Blood	38 ng/mL^†^	Elevated 42–85 ng/mL^†^	8.0 ± 0.2 years	Up to 60 days post-burn	56 ± 0.3^a^
Finnerty *et al.* (2006) [36]	Blood	36.4 ± 9.1 pg/mL	Elevated 118 pg/mL^†^	2–15 years	Immediately after burn	50 ± 3^a^
Jeschke *et al.* (2011) [38]	Blood	160 pg/mL^†^	Within normal limits	7.5 ± 5.3 years	Up to 1100 days post-burn	50 ± 20^b^
**IL-8**						
Jeschke *et al.* (2008a) [37]	Blood	5 ng/mL^†^	Elevated 70–125 ng/mL^†^	8.0 ± 0.2 years	Up to 60 days post-burn	56 ± 0.3^a^
Finnerty *et al.* (2006) [36]	Blood	8.1 ± 3.9 pg/mL	Elevated 40–190 pg/mL^†^	2–15 years	Up to 3 weeks post-burn	50 ± 3^a^
Jeschke *et al.* (2004) [40]	Blood	Not reported	Elevated 300–950 pg/mL^†^	5.7 ± 3.9 years	Up to 40 days post-burn	67 ± 14^b^
Kulp *et al.* (2010) [24]	Urine (in 24 hours)	20 pg/mL^†^	Elevated 90–480 pg/mL^†^	8 ± 5 years	Up to 90 days post-burn	59 ± 17^a^
Jeschke *et al.* (2011) [38]	Blood	<20 pg/mL^†^	Elevated 30–460 pg/mL^†^	7.5 ± 5.3 years	Up to 1100 days post-burn	50 ± 20^b^
**IL-5**						
Jeschke *et al.* (2008a) [37]	Blood	0.65 ng/mL^†^	Reduced 0.3–0.5 ng/mL^†^	8.0 ± 0.2 years	8–10 and 23–29 days post-burn	56 ± 0.3^a^
Jeschke *et al.* (2011) [38]	Blood	1 pg/mL^†^	Elevated 1.5–3.2 pg/mL^†^	7.5 ± 5.3 years	Up to 1100 days post-burn	50 ± 20^b^
**IL-7**						
Jeschke *et al.* (2008a) [37]	Blood	4 ng/mL^†^	Elevated 4.2–5.8 ng/mL^†^	8.0 ± 0.2 years	8–60 days post-burn	56 ± 0.3^a^
Finnerty *et al.* (2006) [36]	Blood	3.3 ± 0.3 pg/mL	Elevated 12–17 pg/mL^†^	2–15 years	Immediately after burn and at 3 weeks post-burn	50 ± 3^a^
Jeschke *et al.* (2011) [38]	Blood	14 pg/mL^†^	Elevated 18–27 pg/mL^†^	7.5 ± 5.3 years	Between 11 and 540 days post-burn	50 ± 20^b^
**IL-10**						
Jeschke *et al.* (2008a) [37]	Blood	1.5 ng/mL^†^	Elevated 3–11.75 ng/mL^†^	8.0 ± 0.2 years	Up to 60 days post-burn	56 ± 0.3^a^
Finnerty *et al.* (2006) [36]	Blood	1.2 ± 0.2 pg/mL	Elevated 78 pg/mL^†^	2–15 years	Immediately after the burn	50 ± 3^a^
Jeschke *et al.* (2004) [40]	Blood	Not reported	Elevated 40–125 pg/mL^†^	5.7 ± 3.9 years	Up to 40 days post-burn	67 ± 14^b^
Jeschke *et al.* (2011) [38]	Blood	8 pg/mL^†^	Elevated 17.5–42.5 pg/mL^†^	7.5 ± 5.3 years	Up to 28 days post-burn	50 ± 20^b^
**G-CSF**						
Jeschke *et al.* (2008a) [37]	Blood	<10 ng/mL^†^	Elevated 40–430 ng/mL^†^	8.0 ± 0.2 years	Up to 60 days post-burn	56 ± 0.3^a^
Finnerty *et al.* (2006) [36]	Blood	Undetectable	Elevated 80–1175 pg/mL^†^	2–15 years	Up to 2 weeks post-burn	50 ± 3^a^
Jeschke *et al.* (2011) [38]	Blood	<10 pg/mL^†^	Elevated 25–1100 pg/mL^†^	7.5 ± 5.3 years	Up to 1100 days post-burn	50 ± 20^b^
Kulp *et* al. (2010) [24]	Urine (in 24 hours)	20 pg/mL	Elevated 50–810 pg/mL	8 ± 5 years	Up to 1105 days post-burn	59 ± 17^a^
**GM-CSF**						
Jeschke *et al.* (2008a) [37]	Blood	Undetectable^†^	Elevated 3–9.8 ng/mL^†^	8.0 ± 0.2 years	Up to 60 days post-burn	56 ± 0.3^a^
Finnerty *et al.* (2006) [36]	Blood	Undetectable	Elevated 9 pg/mL^†^	2–15 years	At 2 weeks post-burn	50 ± 3^a^
Jeschke *et al.* (2011) [38]	Blood	3 pg/mL^†^	Elevated 7.5–23 pg/mL^†^	7.5 ± 5.3 years	Up to 1100 days post-burn	50 ± 20^b^
**IL-4**						
Jeschke *et al.* (2008a) [37]	Blood	Undetectable^†^	Elevated 0.3–1.75 ng/mL	8.0 ± 0.2 years	Up to 60 days post-burn	56 ± 0.3^a^
Finnerty *et al.* (2006) [36]	Blood	Undetectable^†^	Elevated 1.35–2.35 pg/mL^†^	2–15 years	Up to 1 week post-burn	50 ± 3^a^
Jeschke *et al.* (2011) [38]	Blood	<0.5 pg/mL^†^	Elevated 1.5–7.5 pg/mL^†^	7.5 ± 5.3 years	Up to 270 days post-burn	50 ± 20^b^
**IL-13**						
Jeschke *et al.* (2008a) [37]	Blood	0.9 ng/mL^†^	Elevated 1.75–1.9 ng/mL^†^	8.0 ± 0.2 years	Up to 7 days post-burn	56 ± 0.3^a^
Finnerty *et al.* (2006) [36]	Blood	0.7 ± 0.0 pg/mL	Elevated 5.75 pg/mL^†^	2–15 years	Immediately after burn	50 ± 3^a^
Jeschke *et al.* (2011) [38]	Blood	<1 pg/mL^†^	Elevated 2.5–4.8 pg/mL^†^	7.5 ± 5.3 years	Up to 180 days post-burn	50 ± 20^b^
**IL-2**						
Jeschke *et al.* (2008a) [37]	Blood	Undetectable^†^	Elevated 0.4–3.7 ng/mL^†^	8.0 ± 0.2 years	Up to 60 days post-burn	56 ± 0.3^a^
Finnerty *et al.* (2006) [36]	Blood	Undetectable^†^	Elevated 3.75–5.75 pg/mL^†^	2–15 years	Immediately after burn and at 2 weeks post-burn	50 ± 3^a^
Jeschke *et al.* (2011) [38]	Blood	3 pg/mL^†^	Elevated 5–17.5 pg/mL^†^	7.5 ± 5.3 years	Up to 1100 days post-burn	50 ± 20^b^

†Data derived from graph, ^a^Data presented as mean ± SEM, ^b^Data presented as mean ± SD

*TBSA* total body surface area, *IL* interleukin, *TNF* tumour necrosis factor, *INFγ* interferon-gamma, *MCP-1* monocyte chemoattractant protein-1, *MIP-1β* macrophage inflammatory protein 1β, *G-CSF* granulocyte-colony stimulating factor, *GM-CSF* granulocyte-macrophage colony-stimulating factor

#### Initiation of the inflammatory response

Several cytokines involved in the initiation of the inflammatory response are increased following burn injury. IL-17, which is involved in inducing various inflammatory mediators [34] and protecting against microbial infection through stimulating the production of antimicrobial peptides [35], has been reported to increase immediately following a burn [36], and remain elevated for up to 3 years post-burn [37, 38].

Serum abundance of IL-1β, a cytokine that plays a role in the induction of fever and migration of inflammatory cells to the wound site [39], has been observed to be significantly increased at the time of hospital admission in children with burns, compared with healthy children [36, 37]. Over time, IL-1β levels have been observed to decrease in children with burns, although levels remain higher than controls without burns for up to 60 days post-burn [38, 40, 41]. Tumour necrosis factor (TNF) is a cytokine that is often co-expressed with IL-1β and is involved in many of the same processes [42]. There are some discrepancies within the literature about the abundance of TNFα after a burn, as some studies reported no difference in abundance of serum TNFα between children with burns and healthy children [36, 41], while others observed significant increases in TNFα at the time of admission. Multiple studies have reported significant increases in serum TNFα that lasted for up to 1 week post-burn [20, 37], 40 days post-burn [40] and 6 months post-burn [43]. Moreover, TNFα has reportedly remained significantly elevated in urine for up to 180 days compared with healthy children [24]. It is unclear why some studies observed normal levels while others reported prolonged elevation in TNFα but variability in performance of the immunoassay is a possibility [44].

Similarly, IL-6 has exhibited increases of up to 1000-fold in children with burns at hospital admission [41, 45], and remained elevated for months [24, 36, 37, 43, 45] to years [38] after the injury. While most studies observed increases in IL-6 for extended periods of time post-burn, Jeschke et al. (2004) observed elevated levels for only 10 days, and levels returned to normal by day 20 post-burn [40]. This observation is likely due to the accuracy and sensitivity of the biomarker detection platform employed by the authors, as many of the other parameters are comparable between the studies. Authors who used the multiplex Bio-Rad Bio-Plex Suspension Assay reported significantly higher concentrations of IL-6 [24, 36, 37, 43, 45], and longer duration of elevation, than studies that utilized other enzyme-linked immunosorbent assays (ELISA), which may have been less sensitive [20, 40, 41]. It is also important to take into consideration the lower limits of various detection platforms that may give rise to spurious data. IL-6 contributes to acute phase inflammation by stimulating the production of acute phase proteins (e.g. α1-acid glycoprotein, C-reactive protein (CRP), α2-macroglobulin, α 1-antitrypsin and haptoglobin) in the liver [46].

Plasma concentrations of acute phase proteins increase following a burn, decreasing over time back to normal [40]. Alpha-1-acid glycoprotein and C-reactive protein have both been reported to increase immediately following burn injury [20, 37, 41, 47], whereas reported increases in α1-antitrypsin and haptoglobin do not occur until at least 5 days post-burn [38, 40]. According to Jeschke et al. (2008b), the CRP response to burn is significantly lessened in toddlers (0–3.9 years) compared with older children (4–18 years) [48]. This suggests that toddlers exhibit a reduced inflammatory response to burns, as CRP is a well-established marker of acute inflammation [49]. Delayed increases in complement C3 and α2-macroglobulin are reported between 17–60 days and 35–60 days post-burn, respectively [37]. Elevated levels of all these acute phase proteins are reported to persist for at least 2 months post-burn [37, 40, 50]. These proteins have been investigated primarily as markers of the inflammatory response; however, studies outside of burns have hypothesized that prolonged elevation of acute phase proteins may contribute to increased risk of coronary heart disease [51]. Unfortunately, the limited research that exists regarding the role of acute phase proteins in paediatric burns is insufficient to suggest that the response elicited by burn injury in children contributes to this risk. More research is required to fully elucidate the role of acute phase proteins in burn wound healing and determine the potential long-term effects.

Leptin is a hormone most well known for suppressing hunger; however, it also has roles in the inflammatory response, as well as regulating the hypothalamic–pituitary–adrenal axis, angiogenesis, cellular proliferation and nutrient absorption [52]. In paediatric burns, it has been reported to increase at the time of admission [20], and may be involved in the acute phase response [53]. Long-term investigation of this hormone has not been performed and therefore it is unknown how long this hormone remains elevated in children following a burn. Interestingly, leptin levels appear to be correlated with burn size, as participants with burns covering >30% total body surface area (TBSA) had significantly higher levels of leptin than those with smaller burns [20].

IFNγ is involved in the activation of macrophages, inhibition of cell growth, regulation of the production of other inflammatory molecules [54] and the activation of apoptosis in epithelial cells [55]. Studies performed by Finnerty et al. (2006) and Jeschke et al. (2008a) reported increased levels of IFNγ and IL-12p70 (which cross-regulate each other [56]) in children with burns [36, 37]. Conversely, Gauglitz et al. (2009) only reported increased levels of IL-12p70, not IFNγ, in children with burns [43]. Finnerty et al. (2006) observed immediate increases in circulating levels of IFNγ after a burn [36], while Jeschke et al. (2008a) observed elevated IFNγ levels for up to 7 days post-burn, followed by fluctuations of IFNγ until 28 days post-burn [37]. These fluctuations may coincide with other clinical events, such as sepsis; however, this was not explicitly investigated. Gauglitz et al. (2009) observed no difference in IFNγ levels for up to 3 years following a burn; however, acute changes in IFNγ may have been lost during analysis, as the data was separated into broad time points (i.e. >1 month duration) [43].

In burns, procalcitonin (PCT) has been identified by several studies [20, 57–59] and is one of the most well-characterized biomarkers in adult burn research, specifically in terms of its role in predicting sepsis. PCT is the hormone precursor to calcitonin and becomes elevated in response to bacterial infection or inflammation [60]. Although there is less research on PCT in children with burns, the consensus is that PCT increases with burn injury regardless of infection [57]. It has been hypothesized that PCT may be increased as a result of the exacerbated inflammatory response that occurs in response to burn injury as several pro-inflammatory markers are thought to induce PCT secretion [61]. It has been reported that increased levels of PCT are correlated with larger burn size [20]; however, another study failed to find an association between burn size and PCT [58]. In that study, it is possible that any correlation between burn size and PCT may have been confounded by the presence of inhalation injury, which affected 60% of the cohort [58].

#### Mediation of the inflammatory response

There are several markers that are involved in mediating the inflammatory response through the production, activation and regulation of immune cells. Monocyte chemoattractant protein-1 (MCP-1), macrophage inflammatory protein 1β (MIP-1β), also known as CC motif ligand 4 (CCL4) and IL-8 are immune cell chemoattractants that have been reported to increase following a burn [36, 40, 62–64]. Unfortunately, there is little agreement on the duration that each of these markers remains elevated. Several studies suggest that these markers can remain elevated for months [37, 45], and even years [43] after the burn. Other studies have reported elevated levels of MCP-1 lasting only 1 week post-burn and IL-8 for only 3 weeks post-burn [36]. All these studies had similar inclusion criteria, included both genders, analysed the samples using the same method and had similar burn severities, suggesting that other factors are responsible for the difference in the response of MCP-1 and IL-8 to burn injury. Characteristics such as burn mechanism (e.g. flame, scald, etc.) or co-morbidities (such as infection or inhalation injury) may account for the variance [50, 65]. In support of this, another study involving primarily children with flame burns, reported elevated serum IL-8 levels for up to 6 months post-burn, with levels returning to normal by 9 months post-burn [43]. In addition to duration, the magnitude of elevation for each marker following burn injury is of interest. Jeschke et al. (2012a) reported a 100-fold increase in serum MCP-1 immediately following a burn [45]. Notably, the abundance of MCP-1 reported by Finnerty et al. (2006) and Gauglitz et al. (2009), and IL-8 reported by Finnerty et al. (2006) and Jeschke et al. (2004) is three orders of magnitude lower than that reported by Jeschke et al. (2008, 2012) [36, 37, 40, 43, 45] (Table 1). Finally, elevated levels of IL-8 have been observed to coincide with increases of IL-1β, IL-6, IL-10, IL-12p70 and IL-13 in children with burns [50]. This supports the hypothesis that immune function is altered following a burn where both pro-inflammatory and anti-inflammatory markers are elevated simultaneously. This may be due to impairment of the immune system or tight regulation of the system in response to mass insult. In any case, it can increase risk of organ failure and systemic inflammatory response syndrome [50].

Other markers mediate the inflammatory response by alternate means. IL-5 is an inflammatory cytokine involved in white blood cell recruitment, survival and activation [55]; IL-7 is involved in the support and development of T cells during inflammation [66]; IL-10 is involved in preventing over-activation of the immune cells responsible for pathogen clearance [67]; and granulocyte-colony stimulating factor (G-CSF) and granulocyte-macrophage colony-stimulating factor (GM-CSF) are glycoproteins responsible for stimulating the production and release of granulocytes [59, 60], and maintaining granulocyte and macrophage population [61], respectively. Serum levels of IL-5 reported in the literature are contradictory. One study reported that IL-5 levels remained within normal ranges until 8 days post-burn where levels significantly decreased until day 10, returned to normal, then decreased again between days 23 to 29 post-burn [37]. Other studies reported increased IL-5 levels [38]. One study reported elevations that lasted only 1 week [36], while a separate study reported elevations for up to 6 months post-burn, that returned to normal within 9 months post-burn [43]. Interestingly, both studies that reported increased IL-5, primarily investigated flame burns [36, 43] suggesting that burn mechanism may play a role in the IL-5 response to burn injury. IL-7 and IL-10 have been reported to increase immediately following burns in children in one study [36], while another study observed a delayed increase in IL-7 at 8 days post-burn [37]. Both markers are reported to remain higher than normal for at least 60 days post-burn [37], and may remain significantly elevated for up to 3 years post-burn [43]. G-CSF and GM-CSF have been reported to both remain unaffected or to increase after burns. One study reported normal values of G-CSF and GM-CSF within the first 3 years following a burn injury [43], while three other studies have reported increased serum levels of these inflammatory markers that remain elevated for 2 weeks [36], 60 days [37], and 3 years post-burn [38]. Another study that investigated urinary G-CSF observed increased levels for up to 1105 days post-burn [24].

The primary role of IL-4 is in protective immunity against extracellular parasites; however, it also has roles in tissue adhesion and inflammation [55]. One study reported that within 3 years following a burn, serum IL-4 did not significantly differ between children with burns and healthy children [43]. Other studies report significantly elevated levels of IL-4 up to 1 week [36], 60 days [37], or 9 months post-burn [38], although the reported concentration increases differ between the studies. Finnerty et al. (2006) and Jeschke et al. (2011) reported similar values of 1.35–2.35 pg/mL and 1.5–7.5 pg/mL, respectively [36, 38], while Jeschke et al. (2008a) reported values of 0.3–1.75 ng/mL [37]. Furthermore, IL-4 has been shown to exhibit different abundance profiles in females with burns, compared with males, which suggests that the significant differences in age and gender between the burn and control cohorts in the study by Gauglitz et al. (2009) may have influenced their results [43, 68]. Further studies are required to better understand how IL-4 levels change in response to burn injury.

#### Antagonism of the inflammatory response

Several markers are involved in the inflammatory response through antagonizing other cytokines. IL-13 decreases the concentration of pro-inflammatory cytokines and chemokines and produces the IL-1 antagonist, IL-1 receptor α [69]. In paediatric burns, it has been reported to increase immediately following a burn [36], and remain elevated for up to 7 days post-burn [37].

Additionally, IL-2 antagonizes inflammation through interference with pro-inflammatory processes by inhibiting the differentiation of T-helper 17 cells—the cells responsible for producing IL-17 [70]. One study reported that serum IL-2 did not significantly differ between children with burns and healthy children within the first 3 years following burn injury [43]. Conversely, there are other reports of significantly elevated levels of IL-2 [37], lasting for at least 1 week post-burn [36], or up to 3 years post-burn [38]. Although the studies report elevated levels of IL-2, the reported concentration increases of each study differ. While Finnerty et al. (2006) reported values of 3.74–5.75 pg/mL for IL-2 [36], Jeschke et al. (2008a) reported values of 0.4–3.7 ng/mL [37]. Furthermore, significant differences in age and gender between the burn and control groups in the study by Gauglitz et al. (2009) suggest that their results may have been influenced by these factors, as IL-2 has been shown to exhibit different abundance profiles in females with burns, compared with males [43, 68]. More research is required to verify the response of IL-2 to burn injury and provide better understanding of its impact on healing.

### Biomarkers for evaluating tissue repair and/or wound healing

The primary goal for treating children with burns is healing of the wound site. Identifying the markers involved in this healing process is crucial for understanding and predicting the wound healing response. As such, proteins involved in tissue and extracellular matrix (ECM) composition have been evaluated in paediatric burns, along with growth factors that are important for stimulation of wound healing (Table 2).

**Table 2:** Summary of reported abundance of biomarkers involved in tissue repair in children with burns compared to healthy children without burns

**Reference**	**Source**	**Reported normal limits**	**Abundance in children with burns**	**Age range**	**Time frame**	**Population TBSA (%)**
**PICP**						
Klein *et al.* (1995) [41]	Blood	200–700 ng/mL	Normal 210 106 ng/mL	5.8–17.5 years	3 weeks post-burn	63 ± 16^a^
**Collagen (Type IV)**						
Weremijewicz *et al.* (2018) [76]	Blood	50 ng/mL^d†^	Elevated 160–375 ng/mL^d†^	9 months–14 years	From 2 hours post-burn, until at least 5 days post-burn	4–20^e^
**Laminin-5**						
Weremijewicz *et al.* (2018) [76]	Blood	52 ng/mL^d†^	Elevated 72–95 ng/mL^d†^	9 months–14 years	From 2 hours post-burn, until at least 3 days post-burn	4–20^e^
**ProMMP-1**						
Dasu *et al.* (2003) [80]	Blood	3.5 ng/mL^†^	Elevated 15–18 ng/mL^†^	7.9 ± 2.5 years	From 7 days post-burn, until at least 21 days post-burn	>40^c^
**MMP-3**						
Dasu *et al.* (2003) [80]	Blood	110 ng/mL^†^	Elevated 112–130 ng/mL^†^	7.9 ± 2.5 years	From 3 days post-burn, until at least 21 days	>40^c^
**MMP-9**						
Dasu *et al.* (2003) [80]	Blood	350 ng/mL^†^	Elevated 580 ng/mL^†^	7.9 ± 2.5 years	At 21 days post-burn	>40^c^
**TIMP-1**						
Dasu *et al.* (2003) [80]	Blood	250 ng/mL^†^	Elevated 620–700 ng/mL^†^	7.9 ± 2.5 years	From 3 days post-burn, until at least 21 days	>40^c^
**MMP-2**						
Weremijewicz *et al.* (2018) [76]	Blood	38 ng/mL^d†^	Elevated 78–125 ng/mL^d†^	9 months–14 years	From 2 hours post-burn, until at least 5 days post-burn	4–20^e^
**Proteasome**						
Matuszczak *et al.* (2014) [82]	Blood	0.42 U/mg^†^	Elevated 0.75–1.3 U/mL^†^	9 months–14 years	At 12–16 hours post-burn	4–20^e^
**Hepatocyte growth factor**						
Jeschke *et al.* (2004) [40]	Blood	0.5 ± 0.2 ng/mL	Elevated 1.75–2.25 ng/mL^†^	1–16 years	Immediately after burn, until 15 days post-burn	67 ± 14^b^
**TGFα**						
Abdel-Hafez *et al.* (2007) [20]	Blood	8.08 ± 1.66 pg/mL	Elevated 170.81 ± 16.65 pg/mL	2 months–7 years	At admission	31.62 ± 12.01^b^
**TGFβ**						
Rorison *et al.* (2010) [21]	Blood	420 pg/mL^d^	Reduced 280 pg/mL^d^	3.82 ± 3.55 years	At admission	9.1 ± 11.7^b^
**bFGF**						
Abdel-Hafez *et al.* (2007) [20]	Blood	0.56 ± 0.13 ng/mL	Elevated 0.98 ± 0.22 ng/mL	2 months–7 years	At admission	31.62 ± 12.01^b^

†Data derived from graph, ^a^Data presented as mean ± SEM, ^b^Data presented as mean ± SD, ^c^Data presented as minimum value, ^d^Data presented as median, ^e^Data presented as a range

*TBSA* total body surface area, *PICP* carboxyterminal propeptide of type I procollagen, *MMP* matrix metalloproteases, *TIMP-1* tissue inhibitor of metalloproteinases-1, *TGF* transforming growth factor, *bFGF* basic fibroblast growth factor

#### Structural proteins

In general, structural proteins have been observed to increase following a burn in children, except for the carboxyterminal propeptide of type I procollagen (PICP), pyridinoline and deoxypyridinoline. PICP is an indicator of type I collagen synthesis, which is required for formation of connective tissue, including bone and skin [71, 72]. Pyridinoline and deoxypyridinoline, the collagen fibre crosslinks in bone, are markers of bone resorption [73]. In a study conducted by Klein et al. (1995), mean levels of PICP, urinary pyridinoline and urinary deoxypyridinoline were reduced when assessed at several time points across the first 2 to 3 weeks in children who sustained a burn [41]. This may contribute to the hypothesized decrease in bone formation or repair following burn injury [74]. Other structural proteins, such as Type IV collagen (the most abundant collagen in basement membranes [75]) and laminin-5 (a basement membrane glycoprotein that promotes epithelial cell anchorage) have been observed to increase. In children, collagen IV increases immediately after a burn injury, peaking at 12–16 hours after the burn, then returns to normal levels within 5 days post-burn [76]. Furthermore, laminin-5 is significantly elevated in the blood of children with burns for up to 3 days post-injury, compared with healthy controls [76]. This elevation may be due to liberation of the protein through the destruction of the basement membrane by the burn. Alternatively, it may be an indicator of wound healing, as laminin-5 also facilitates the cellular adhesion and migration of keratinocytes, along the basement membrane [77]. Matrix metalloproteases (MMPs) are involved in tissue remodelling through the degradation of ECM and help to mediate biological processes such as inflammation, bone remodelling and angiogenesis [78]. Typically, MMPs increase in wounds as they are essential for breaking down the wound bed, allowing for wound healing and scar formation [79]. It is thought that IL-17 may play a role in tissue healing after injury, through the promotion of keratinocyte proliferation, or in scarring, through the stimulation of MMP production [35]. One study from 2003 identified increased levels of ProMMP-1, MMP-3 and MMP-9 within the first 3 weeks of burn injury [80], while a second study found significant elevations in MMP-2 for the first 5 days post-burn [76]. Tissue inhibitor of metalloproteinases-1 (TIMP-1) has also been found to be significantly elevated at 3, 7 and 21 days post-burn compared with healthy controls [80]. It is thought that the ratio of MMPs to TIMPs determines whether beneficial wound repair is achieved. While multiple studies have investigated MMPs in paediatric burns, Dasu and colleagues are the first and only group to evaluate TIMP-1 [80]. Based on their findings, they hypothesized that TIMP-1, in conjunction with MMPs, has a beneficial role in wound healing following a burn; however, more research is required to verify this. Finally, in burns where extensive tissue damage is present, the activity of proteasomes (protein complexes that degrade damaged proteins) [81], has been reported to be elevated compared with healthy controls [82]. These elevated levels were also negatively correlated with total protein levels in blood. Circulating proteasome levels were also correlated with burn severity, suggesting that more severe burns have increased levels of catabolism. This could be solely due to an increased amount of damaged proteins that need to be cleared or could be an indicator of more severely altered systemic metabolism as a result of the burn injury.

#### Growth factors involved in healing

Many growth factors play a role in tissue repair and wound healing. Hepatocyte growth factor contributes to wound healing through the promotion of motility and morphogenesis of epithelial cells, while also playing a major role in angiogenesis [83]. It has been reported to increase in paediatric burns and remain elevated for at least 2 weeks post-burn [40]. In paediatric burns, Abdel-Hafez et al. (2007) reported elevated levels of TGFα, a growth factor that influences cellular migration, cellular proliferation and angiogenesis [84], at the time of admission [20]. Although it is often described as an immune modulator [85], TGFβ_1_ has many roles within the body including cellular differentiation, immune regulation and wound healing [86]. Specifically in burns, TGFβ_1_ has been associated with collagen production and scar formation during the scarring process and may be of less importance during acute phase healing [87, 88]. TGFβ_1_, in its active form, has been observed to be significantly lower in children with burns than healthy controls on the day of the burn [21], whereas total TGFβ_1_ was reported to be not significantly different between children with burns and healthy children without burns. Basic fibroblast growth factor (bFGF) is also involved in repair and regeneration of tissue [89]. In children, only one study has evaluated endogenous levels of bFGF following burn injury, whereby serum levels of bFGF were reported to increase at the time of admission [20]. Other studies have evaluated the effect of topical bFGF on scar outcome in children and reported improved healing outcomes [90, 91]. It is important to understand how the topical application of bFGF alters the healing outcome and therefore more research is required to elucidate the specific role that bFGF plays in paediatric burn wound healing.

### Biomarkers for evaluating changes to growth and metabolism

Burn injury is known to alter metabolism which can lead to stunted growth in children [92]. Therefore, by understanding how burns affect these processes, any alterations in growth can be monitored and treated, or prevented, before they have serious impacts on the child’s development. Consequently, markers involved in metabolism have been evaluated in paediatric burns (Table 3).

**Table 3:** Summary of reported abundance of biomarkers associated with growth and metabolism in children with burns compared to healthy children without burns

**Reference**	**Source**	**Reported normal limits**	**Abundance in children with burns**	**Age range**	**Time frame**	**Population TBSA (%)**
**Growth hormone**						
Jeschke *et al.* (2008a) [37]	Blood	4 ng/mL^†^	Reduced 1.1–2.8 ng/mL^†^	8.0 ± 0.2 years	8 to 60 days post-burn	56 ± 0.3^a^
Fleming *et al.* (1992) [95]	Blood	<8 ng/mL	Within normal limits 2.3 ± 0.3 ng/mL	11.1 ± 1.4 years	2 to 3 weeks post-burn	67 ± 6^a^
Gauglitz *et al.* (2009) [43]	Blood	3.92 ± 5.23 ng/mL	Reduced 0.86 ± 1.50–1.74 ± 1.10 ng/mL	8.8 ± 5.3 years	Up to 36 months post-burn	57.9 ± 14.7^b^
Jeschke *et al.* (2011) [38]	Blood	4.5 ng/mL^†^	Reduced 1.75–2.75 ng/mL^†^	7.5 ± 5.3 years	Sporadically over 1100 days post-burn	50 ± 20^b^
**IGF-1**						
Jeschke *et al.* (2008a) [37]	Blood	225 ng/mL^†^	Reduced 25–45 ng/mL^†^	8.0 ± 0.2 years	Up to 60 days post-burn	56 ± 0.3^a^
Jeschke *et al.* (2004) [40]	Blood	365 ± 15 μg/mL	Reduced 92 ± 36–147 ± 42 μg/mL	5.7 ± 3.9 years	Up to 40 days post-burn	67 ± 14^b^
Fleming *et al.* (1992) [95]	Blood	22–138 U/mL	Within normal limits 56 ± 15 U/mL	11.1 ± 1.4 years	2 to 3 weeks post-burn	67 ± 6^a^
Gauglitz *et al.* (2009) [43]	Blood	183 ± 178.22 ng/mL	Reduced 72.01 ± 60.51–124.97 ± 126.23 ng/mL	8.8 ± 5.3 years	Up to 2 months post-burn	57.9 ± 14.7^b^
Jeschke *et al.* (2011) [38]	Blood	175 ng/mL^†^	Reduced 30–120 ng/mL^†^	7.5 ± 5.3 years	Up to 270 days post-burn	50 ± 20^b^
**IGFBP-3**						
Jeschke *et al.* (2008a) [37]	Blood	3800 ng/mL^†^	Reduced 1100–1900 ng/mL^†^	8.0 ± 0.2 years	Up to 60 days post-burn	56 ± 0.3^a^
Jeschke *et al.* (2004) [40]	Blood	2.8 ± 0.9 μg/mL	Reduced 0.6 ± 0.2–1.0 ± 0.4 μg/mL	5.7 ± 3.9 years	Up to 40 days post-burn	67 ± 14^b^
Gauglitz *et al.* (2009) [43]	Blood	3788.04 ± 1391.14 ng/mL	Reduced 1752.32 ± 978.80–2289.49 ± 1503.46 ng/mL	8.8 ± 5.3 years	Up to 2 months post-burn	57.9 ± 14.7^b^
Jeschke *et al.* (2011) [38]	Blood	4100 ng/mL^†^	Reduced 1250–3400 ng/mL^†^	7.5 ± 5.3 years	Up to 1100 days post-burn	50 ± 20^b^
**IGFBP-1**						
Jeschke *et al.* (2004) [40]	Blood	115 ± 15 μg/mL	Elevated 170 ± 100 μg/mL	5.7 ± 3.9 years	At admission	67 ± 14^b^
**β-Estradiol (oestrogen)**						
Jeschke *et al.* (2008a) [37]	Blood	70 ng/mL^†^	Reduced 23–38 ng/mL^†^	8.0 ± 0.2 years	Immediately after the burn and between 11- and 28-days post-burn	56 ± 0.3^a^
Jeschke *et al.* (2011) [38]	Blood	77.5 pg/mL^†^	Reduced 20–45 pg/mL^†^	7.5 ± 5.3 years	Up to 1100 days post-burn	50 ± 20^b^
**Testosterone**						
Jeschke *et al.* (2008a) [37]	Blood	110 ng/mL^†^	Reduced 40–42 ng/mL^†^	8.0 ± 0.2 years	At 29–60 days post-burn	56 ± 0.3^a^
Jeschke *et al.* (2011) [38]	Blood	110 ng/mL^†^	Elevated 180 ng/mL^†^	7.5 ± 5.3 years	At 8–10 days post-burn	50 ± 20^b^
Jeschke *et al.* (2011) [38]	Blood	110 ng/mL^†^	Reduced 40–45 ng/mL^†^	7.5 ± 5.3 years	At 61–90 days and 271–365 days post-burn	50 ± 20^b^
**Progesterone**						
Jeschke *et al.* (2008a) [37]	Blood	60 ng/mL^†^	Elevated 125–230 ng/mL^†^	8.0 ± 0.2 years	Up to 7 days post-burn, between days 11 and 28 post-burn and at 35–60 days post-burn.	56 ± 0.3^a^
Jeschke *et al.* (2011) [38]	Blood	60 ng/mL^†^	Elevated 100–200 ng/mL^†^	7.5 ± 5.3 years	Up to 540 days post-burn	50 ± 20^b^
**Insulin**						
Jeschke *et al.* (2008a) [37]	Blood	15 ng/mL^†^	Elevated 40–160 ng/mL^†^	8.0 ± 0.2 years	Up to 60 days post-burn	56 ± 0.3^a^
Fleming *et al.* (1992) [95]	Blood	5–25 μU/mL	Within normal limits 25.0 ± 3.0 μU/mL	11.1 ± 1.4 years	2 to 3 weeks post-burn	67 ± 6^a^
Gauglitz *et al.* (2009) [43]	Blood	8.1 μIU/mL^†^	Elevated 11–13.5 μIU/mL^†^	8.8 ± 5.3 years	From 6 months post-burn, up to 36 months post-burn	57.9 ± 14.7^b^
Jeschke *et al.* (2012a) [45]	Blood	8 μIU/mL^†^	Elevated 38–75 μIU/mL^†^	8 ± 5 years	Up to 250 days post-burn	64 ± 12^b^
Gottschlich *et al.* (2002) [103]	Blood	0–30 μIU/mL	Elevated 69–40 μIU/mL	9.6 ± 0.7 years	From 2 weeks up to 4 weeks post-burn	53.2 ± 3.4^a^
Fram *et al.* (2010) [105]	Blood	7.4 ± 1.0 μIU/mL	Elevated 16.6 ± 7.8 μIU/mL	8 ± 4.6 years	At time of 95% re-epithelialization (67.9 ± 15 days)	66 ± 15^a^
Jeschke *et al.* (2011) [38]	Blood	<10 IU/mL^†^	Elevated 15–108 IU/mL^†^	7.5 ± 5.3 years	Up to 1100 days post-burn	50 ± 20^b^
**C-peptide**						
Gauglitz *et al.* (2009) [43]	Blood	0.6 ng/mL	Elevated 0.95–1.25 ng/mL	8.8 ± 5.3 years	Up to 36 months post-burn	57.9 ± 14.7^b^
**Glucose**						
Jeschke *et al.* (2008a) [37]	Blood	90 mg/dl^†^	Elevated 125–170 mg/dl^†^	8.0 ± 0.2 years	Up to 60 days post-burn	56 ± 0.3^a^
Jeschke *et al.* (2012a) [45]	Blood	85 mg/dl^†^	Elevated 122–155 mg/dl^†^	8 ± 5 years	Up to 250 days post-burn	64 ± 12^b^
Fleming *et al.* (1992) [95]	Blood	60–115 mg/dl	Elevated 129 ± 13 mg/dl	11.1 ± 1.4 years	2 to 3 weeks post-burn	67 ± 6^a^
Gauglitz *et al.* (2009) [43]	Blood	83 mg/dl^†^	Elevated 94–90 mg/dl^†^	8.8 ± 5.3 years	Up to 6 months post-burn	57.9 ± 14.7^b^
Fram *et al.* (2010) [105]	Blood	73.6 ± 1.3 mg/dl	Elevated 92.3 ± 4.5 mg/dl	8 ± 4.6 years	At time of 95% re-epithelialization (67.9 ± 15 days)	66 ± 15^a^
Gottschlich *et al.* (2002) [103]	Blood	60–105 mg/dl	Elevated 123–153 mg/dl	9.6 ± 0.7 years	Up to 4 weeks post-burn	53.2 ± 3.4^a^
Jeschke *et al.* (2011) [38]	Blood	90 mg/dl^†^	Elevated 110–160 mg/dl^†^	7.5 ± 5.3 years	Up to 180 days post burn	50 ± 20^b^
**Free fatty acids**						
Jeschke *et al.* (2004) [40]	Blood	0.3 ± 0.05 μEq/l	Elevated 0.55–0.68 μEq/L^†^	1–16 years	Immediately after burn until 5 days post-burn	67 ± 14^b^
Jeschke *et al.* (2008a) [37]	Blood	0.4 ng/mL^†^	Elevated 0.57–1.13 ng/mL^†^	8.0 ± 0.2 years	Immediately after burn, then from 8–34 days post-burn	56 ± 0.3^a^
Fleming *et al.* (1992) [95]	Blood	0.19–0.9 mEq/L	Within normal limits 0.59 ± 0.04 mEq/L	11.1 ± 1.4	2 to 3 weeks post-burn	67 ± 6^a^
**Triglycerides**						
Jeschke *et al.* (2004) [40]	Blood	110 ± 13 mg/dl	Elevated 155–245 mg/dl^†^	1–16 years	From 10 to 80 days post-burn	67 ± 14^b^
Jeschke *et al.* (2008a) [37]	Blood	85 ng/mL^†^	Elevated 130–195 ng/mL^†^	8.0 ± 0.2 years	Up to 60 days post-burn	56 ± 0.3^a^
Jeschke *et al.* (2011) [38]	Blood	110 mg/dL^†^	Elevated 165–210 mg/dL^†^	7.5 ± 5.3 years	Between 17 days and 180 days post-burn	50 ± 20^b^
**T3**						
Gottschlich *et al.* (2002) [103]	Blood	125–250 μg/dl	Reduced 35.7–63.8 ng/dl	9.6 ± 0.7 years	Up to 4 weeks post-burn	53.2 ± 3.4^a^
**T4**						
Jeschke *et al.* (2008a) [37]	Blood	8.5 ng/mL^†^	Reduced 4.5–7.5 ng/mL^†^	8.0 ± 0.2 years	Up to 60 days post-burn	56 ± 0.3^a^
Gottschlich *et al.* (2002) [103]	Blood	6–12.5 ng/dl	Reduced 3.62–4.10 ng/dl	9.6 ± 0.7 years	Up to 2 weeks post-burn	53.2 ± 3.4^a^
**Albumin**						
Klein *et al.* (1995) [41]	Blood	35.0 ± 55.0 g/L	Reduced 22.8 ± 3.7 g/L	5.8–17.5 years	3 weeks post-burn	63 ± 16^a^
Palmieri *et al.* (2006) [115]	Blood	3.5–4.8 mg/dl	Reduced 2.2 ± 0.2 mg/dl	0–17 years	At admission	41.8 ± 3.8^a^
Gottschlich *et al.* (2002) [103]	Blood	3.2–5.7 g/dl	Reduced 2.2–2.3 g/dl	9.6 ± 0.7 years	Up to 4 weeks post-burn	53.2 ± 3.4^a^
Jeschke *et al.* (2011) [38]	Blood	4.7 g/dl^†^	Reduced 2.3–4 g/dl^†^	7.5 ± 5.3 years	Up to 1100 days post-burn	50 ± 20^b^
**Pre-albumin**						
Jeschke *et al.* (2004) [40]	Blood	35 ± 5 mg/dl	Reduced 7.5–22 mg/dl^†^	1–16 years	Up to 80 days post-burn	67 ± 14^b^
Jeschke *et al.* (2008a) [37]	Blood	19 ng/mL^†^	Reduced 8–16.5 ng/mL	8.0 ± 0.2 years	Up to 60 days post-burn	56 ± 0.3^a^
Gottschlich *et al.* (2002) [103]	Blood	9.5–46.6 mg/dl	Reduced 7.6–7.9 mg/dl	9.6 ± 0.7 years	Up to 2 weeks post-burn	53.2 ± 3.4^a^
Jeschke *et al.* (2011) [38]	Blood	17 mg/dL^†^	Reduced 7.5–13.5 mg/dL^†^	7.5 ± 5.3 years	Up to 28 days post-burn	50 ± 20^b^
**Transferrin**						
Jeschke *et al.* (2004) [40]	Blood	310 ± 50 mg/dl	Reduced 75–165 mg/dl^†^	1–16 years	Up to 80 days post-burn	67 ± 14^b^
Jeschke *et al.* (2008a) [37]	Blood	235 ng/mL^†^	Reduced 90–130 ng/mL^†^	8.0 ± 0.2 years	Up to 60 days post-burn	56 ± 0.3^a^
Gottschlich *et al.* (2002) [103]	Blood	118–328 mg/dl	Reduced 89–109 mg/dl	9.6 ± 0.7 years	Up to 2 weeks post-burn	53.2 ± 3.4^a^
Jeschke *et al.* (2011) [38]	Blood	215 mg/dL^†^	Reduced 80–150 mg/dL^†^	7.5 ± 5.3 years	Up to 90 days post-burn	50 ± 20^b^
**Retinol binding protein**						
Jeschke *et al.* (2004) [40]	Blood	5.0 ± 0.2 mg/dl	Reduced 1.2–3.75 mg/dl^†^	1–16 years	Up to 15 days post-burn	67 ± 14^b^
Jeschke *et al.* (2008a) [37]	Blood	3.5 ng/mL^†^	Reduced 1.8–3.2 ng/mL^†^	8.0 ± 0.2 years	Up to 60 days post-burn	56 ± 0.3^a^
Gottschlich *et al.* (2002) [103]	Blood	3–6 mg/dl	Reduced 1.25–1.92 mg/dl	9.6 ± 0.7 years	Up to 2 weeks post-burn	53.2 ± 3.4^a^
Jeschke *et al.* (2011) [38]	Blood	2.5 mg/dL^†^	Reduced 0.8–1.4 mg/dL^†^	7.5 ± 5.3 years	Up to 10 days post-burn	50 ± 20^b^
Jeschke *et al.* (2011) [38]	Blood	2.5 mg/dL^†^	Elevated 3.3–3.4 mg/dL^†^	7.5 ± 5.3 years	Between days 61 and 180 post-burn	50 ± 20^b^
**Parathyroid hormone**						
Jeschke *et al.* (2008a) [37]	Blood	90 ng/mL^†^	Reduced 8–18 ng/mL^†^	8.0 ± 0.2 years	Up to 60 days post-burn	56 ± 0.3^a^
Jeschke *et al.* (2011) [38]	Blood	85 pg/mL^†^	Reduced 10–25 pg/mL^†^	7.5 ± 5.3 years	Up to 1100 days post-burn	50 ± 20^b^
**Osteocalcin**						
Jeschke *et al.* (2008a) [37]	Blood	52 ng/mL^†^	Reduced 8–18 ng/mL^†^	8.0 ± 0.2 years	Up to 60 days post-burn	56 ± 0.3^a^
Jeschke *et al.* (2011) [38]	Blood	55 ng/mL^†^	Reduced 12.5–42 ng/mL^†^	7.5 ± 5.3 years	Up to 270 days post-burn	50 ± 20^b^
**Apolipoprotein A1**						
Jeschke *et al.* (2008a) [37]	Blood	115 ng/mL^†^	Reduced 50–75 ng/mL^†^	8.0 ± 0.2 years	Up to 60 days post-burn	56 ± 0.3^a^
Jeschke *et al.* (2011) [38]	Blood	105 mg/dL^†^	Reduced 50–80 mg/dL^†^	7.5 ± 5.3 years	Up to 90 days post-burn	50 ± 20^b^
**Apolipoprotein B**						
Jeschke *et al.* (2008a) [37]	Blood	130 ng/mL^†^	Reduced 80–115 ng/mL^†^	8.0 ± 0.2 years	Immediately after burn until 7 days post-burn	56 ± 0.3^a^
Jeschke *et al.* (2008a) [37]	Blood	130 ng/mL^†^	Elevated 150–170 ng/mL^†^	8.0 ± 0.2 years	Around day 23 to day 60 post-burn	56 ± 0.3^a^
Jeschke *et al.* (2011) [38]	Blood	75 mg/dL^†^	Reduced 50–70 mg/dL^†^	7.5 ± 5.3 years	Up to 22 days post-burn	50 ± 20^b^
Jeschke *et al.* (2011) [38]	Blood	75 mg/dL^†^	Elevated 87.5–90 mg/dL^†^	7.5 ± 5.3 years	Between 41 and 90 days post-burn	50 ± 20^b^

†Data derived from graph, ^a^Data presented as mean ± SEM, ^b^Data presented as mean ± SD

*TBSA* total body surface area, *IGF* insulin-like growth factor, *IGFBP* insulin-like growth factor binding protein, *T3* triiodothyronine, *T4* thyroxine

#### Biomarkers involved in growth and development

Growth hormone (GH) is a peptide hormone that is involved in many biological activities that foster growth and metabolism [93], primarily through stimulation of insulin-like growth factor (IGF) [94]. In paediatric burns research, GH has most commonly been reported to be reduced. For instance, Jeschke et al. (2008a) reported a delayed decrease in GH at 8 days post-burn that remained reduced for up to 60 days post-burn [37]. Furthermore, Gauglitz et al. (2009) reported significantly decreased serum levels of GH for up to 3 years post-burn [43]. Conversely, Fleming et al. (1992) reported that GH was within normal limits in children with burns at 2 to 3 weeks post-burn; however, the reported normal value of GH in this study was <8 ng/mL, whereas most other studies report normal levels at 4 ng/mL [95]. GH abundance has also been reported to be affected by age, whereby toddlers (aged 0–3.9 years) had higher levels of GH between 2 and 7 days post-burn, compared with older children [48]. GH is regulated by circadian rhythm, where levels peak shortly after falling asleep [96]. Variations in the reported levels may be a result of inconsistent sampling times within and across studies. Alternatively, disturbances in sleep patterns during hospitalization may explain the reduced values of GH observed in paediatric burns [97].

Insulin-like growth factor-1 (IGF-1) is a peptide hormone that acts systemically to coordinate balanced growth and locally to facilitate processes such as wound healing [98]. It exists in serum, bound to IGF binding proteins [99], such as IGF binding protein 3 (IGFBP-3). IGFBP-3 binds more than 75% of available IGF-1 and transports IGF-1 as well as enhancing their combined half-lives [98]. The general consensus is that IGF-1 and IGFBP-3 decrease following burn injury and can remain reduced for months [37, 38, 40, 43]. It has been reported that IGF-1 levels increase over time [100], and return to normal levels by 9 months post-injury [43]. In contrast to this, one study has reported normal IGF-1 levels in children with burns [95]. However, that study by Fleming et al. (1992) reported the levels of IGF-1 in terms of activity (U/mL) compared with other studies that report IGF-1 abundance (ng/mL), which makes it difficult to comment on the difference in results obtained by these studies. IGFBP-1, another binding protein of IGF-I, has also been investigated in paediatric burns and was observed to increase at the time of admission to hospital [40]. This supports the idea that burns elicit a hypermetabolic state as IGFBP-1 is known to be upregulated in catabolic states [101]. Decreased levels of IGF-1, as well as GH, may play a role in the delayed growth observed in children following a burn injury [102].

Sex hormones such as oestrogen, testosterone and progesterone have been investigated in burns. Estradiol is the primary oestrogen sex hormone and has been reported to decrease following a burn [37, 38]. In adolescents with burns, oestrogen has been observed not to decrease, and remain at a much higher level compared with younger children [48]. This may be due to already high levels of oestrogen being present prior to sustaining the burn. Testosterone was observed at normal levels in a cohort of children with burns, until 4 weeks post-burn when testosterone significantly decreased [37]; however, the cohort was not stratified by sex [38]. This is important because another study reported that testosterone significantly increases immediately post-burn in males [68]. A transient increase in testosterone levels was also observed in another study at 8–10 days post-burn [38]. Progesterone has been reported to be elevated for up to 1 week post-burn [37], then levels appeared to fluctuate, with levels increasing between 11 and 28 days post-burn, and again at 35–60 days post-burn [37]. In a separate study, progesterone was reported to remain increased for up to 540 days post-burn [38].

#### Biomarkers involved in energy metabolism

Energy production is a crucial process within the body that can be altered in children with burns. Several markers involved in different processes of energy metabolism have been investigated. The levels of energy precursors, such as glucose, free fatty acids (FFA) and triglycerides, as well as hormones involved in energy metabolism, such as insulin, triiodothyronine (T3) and thyroxine (T4), have been evaluated in paediatric burns. The hormone insulin, which helps the cells to absorb glucose, has been reported to increase within the first few weeks after a burn [37, 45, 103]. Similarly, C-peptide, a peptide cleaved from proinsulin during the production of insulin [104], has also been reported to increase post-burn in children [43], suggesting that burn injury stimulated the production of insulin. Insulin has otherwise been reported to remain within normal limits immediately following a burn [95], with a delayed elevation at 6 months post-burn lasting for up to 3 years post-burn [43]. These results may be due to the time points selected for the studies. Fleming et al. (1992) collected blood between 2 and 3 weeks post-burn and Gauglitz et al. (2009) collated their data as mean abundance over 1 month periods, potentially resulting in the authors missing the insulin increase following a burn or skewing the data [43, 95]. Additionally, Gauglitz et al. (2009) recruited obese or potentially diabetic children without burns as controls, which may not reflect a true healthy population [43]. Although, some studies have observed elevated levels of insulin persisting for months after the burn injury [37, 45]. In fact, Fram et al. (2010) reported elevated levels of insulin at the time of 95% re-epithelialization, which was 67.9 ± 15 days post-burn [105]. Furthermore, C-peptide has been reported to remain elevated for as long as 3 years post-burn [43]. As insulin enables cells to absorb glucose, it follows that any increase in insulin will correspond with a decrease in serum glucose. In general, fasting serum glucose in children with burns is elevated immediately post-burn [43, 45, 95, 105] and can remain elevated for up to 60 days post-burn [37] or until the burn has reached 95% re-epithelialization (67.9 ± 15 days post-burn) [105]. Serum glucose then decreases over time [45, 103]. Gauglitz et al. (2009) reported that glucose returns to normal around 6 months post-burn [43]. Derangement in glucose homeostasis is evident following burn injury as glucose levels increase and decrease irrespective of insulin control, potentially leading to profound insulin resistance [45].

FFA and triglycerides are also affected by thermal injury and as a result have been investigated in paediatric burns. FFA increase following a burn injury and remain elevated for up to 5 days post-burn [40]. One study observed increased levels of FFA for up to 34 days post-burn [37], whereas a separate study found at 2 to 3 weeks post-burn, FFA were within normal limits [95]. These conflicting results are most likely due to the reported normal limits, as Fleming et al. (1992) reported normal values between 0.19–0.9 mEq/L [95] whereas Jeschke et al. (2004) reported normal values as 0.3 μEq/L [40]. Furthermore, another study by Jeschke et al. (2008a) reported normal values as 0.4 ng/mL [37]. The inconsistencies between reported normal values may be due to the specific characteristics of the control population, particularly the weight of the participants. However, weight or BMI are not reported by every study, which makes it very difficult to accurately compare these results. Increased levels of FFA in children with burns have been associated with elevated levels of α2-macroglobulin [106]. Furthermore, it has been reported that females exhibit significantly lower FFA after 21 days post-burn [68]. This is concordant with previous reports that suggest oestrogen has an effect on lipolysis and blood levels of FFA [107]. Triglycerides (TG) have also been reported to increase following a burn. Initial studies reported a delayed increase in serum TGs occurring at 10 days post-burn [40], whereas a more recent study observed immediate increases in TGs [37]. Both studies reported that TGs remained elevated for at least 2 months post-burn. High levels of TG in children with burns have been accompanied by increased levels of CRP, retinol binding protein and complement C3, compared with children with burns who had normal levels of TG [106].

T3 and T4 are thyroid hormones that are involved in the maintenance of metabolic processes in the body [108]. Both hormones have been reported to decrease immediately after a burn [103], then increase over time [109]. T4 (the less biologically active precursor to T3) has been reported to remain decreased for up to 60 days post-burn [37]. T3 is produced in the periphery by enzymatic cleavage of its precursor [110] and has been reported to remain lower than normal for up to 4 weeks post-burn [103]. Interestingly, females have been observed to have higher levels of T4 at 12 months post-burn, compared with males [109].

#### Other regulatory markers

Biomarkers associated with several different homeostatic mechanisms have been investigated in children with burns, including proteins involved in blood transport, calcium and cholesterol homeostasis.

##### Constitutive hepatic proteins

Constitutive hepatic proteins, including albumin, prealbumin, transferrin and retinol binding protein (RBP) are blood transport proteins that are important for maintaining homeostatic processes [111]. Under stressful and inflammatory conditions, including following burn injury, constitutive protein synthesis is downregulated to allow for the upregulation of acute phase proteins by the liver [111–113]. Albumin, the most abundant serum protein, is a carrier protein for fatty acids, hormones, drugs and metabolites [114], and has been reported at reduced levels at the time of admission [115], at 3 weeks post-burn [41], and for up to 3 years after burn injury [38]. Prealbumin, another serum protein, is significantly reduced following burn injury in children [37, 103]. One study reported decreased levels of prealbumin for up to 80 days post-burn [40]. Interestingly, males have a more profound decrease in prealbumin after burn injury than females [68]. Prealbumin is primarily a carrier protein [116] that is regulated by the acute phase response as well as neuroendocrine changes, and has a gender-specific response to trauma [117]. Transferrin, a free peptide that is primarily involved in iron metabolism through binding iron and transporting it between sites of absorption, utilization, storage and degradation [118], has been observed to be decreased within the first 2 weeks after injury [103]. Belmonte et al. (1999) reported that during the first 48–72 hours (acute stress phase), transferrin was significantly lower than during the recovery phase (17.8 ± 7.4 days post-burn) [47]. Conversely, several studies by Jeschke et al. (2008, 2004 and 2011) have observed decreased serum levels of transferrin for up to 60 days [37], 80 days [40] and 3 months post-burn [38]. Age related differences in abundance have been observed whereby prepubescent children (4–9.9 years) exhibited significantly higher transferrin levels than adolescents (10–18 years) [48]. Retinol binding protein decreases following a burn, thereby reducing its normal action to transport Vitamin A [119] and potentially influencing insulin resistance [120]. Jeschke et al. (2004) and Gottschlich et al. (2002) have reported decreased levels for up to 2 weeks post-burn [40, 103], whereas Jeschke et al. (2008a) reported reduced levels for up to 60 days post-burn [37]. Interestingly, children with burns who exhibited high RBP have also been observed to have significantly increased levels of IL-6, IL-8, MCP-1, osteocalcin, prealbumin, and triglycerides compared with children with burns who exhibited low RBP [121]. This suggests that RBP may also play a role in inflammation, bone catabolism and lipolysis.

##### Calcium homeostasis

In burns, parathyroid hormone (PTH) and osteocalcin have been reported to be reduced for up to 3 years and 270 days post-burn, respectively [37, 38]. PTH is an endocrine regulator of calcium homeostasis [122] and osteocalcin is a protein hormone secreted by osteoblasts that has a role in regulating bone matrix mineralization [123]. Decline in the production of these hormones may be responsible for the increased risk of bone fracture and stunted growth that has been reported following burns in children [124].

##### Cholesterol homeostasis

Apolipoproteins are transport proteins for cholesterol and lipids [125] and apolipoprotein A1 is specifically involved in high-density lipoprotein structure and cholesterol homeostasis [126]. In paediatric burns, apolipoprotein A1 has been reported to decrease in response to a burn, and remain reduced for up to 60 days post-burn [37]. Apolipoprotein B, which is involved in the formation and metabolism of low-density lipoproteins [127], has been reported to decrease immediately following a burn, remaining low for up to 7 days post-burn. This decrease is then followed by a significant increase around 3 weeks post-burn [37] where it remains higher than normal for up to 3 months post-burn [38]. Interestingly, in children with burns who exhibited increased levels of FFA and TG, apolipoprotein B levels were also observed to be elevated [106].

### Biomarkers for evaluating stress

Burn injuries are a complex form of trauma as they consist of both a physical trauma (i.e. the burn) and a psychological trauma (as reviewed by De Sousa (2010) [128]). Early identification of stress and trauma in children with burns is of great importance, as increased stress experienced by a child in the initial stages can predispose them to more severe psychological issues later in life [129]. A study investigating the incidence of adverse psychological outcomes in adults with a history of childhood burns has confirmed that burn-related stress in childhood can result in the development of suicidal ideation, anxiety disorders and depressive disorders [130]. Stoddard et al. (2017) found that in a population of children younger than 4 years who sustained a burn, 10% met full diagnostic criteria for PTSD just 1 month after the injury and another 27% met partial diagnostic criteria for PTSD [131]. Through monitoring of biological markers to identify stress early, interventions may be put in place to ameliorate effects into the future.

**Figure 2.**
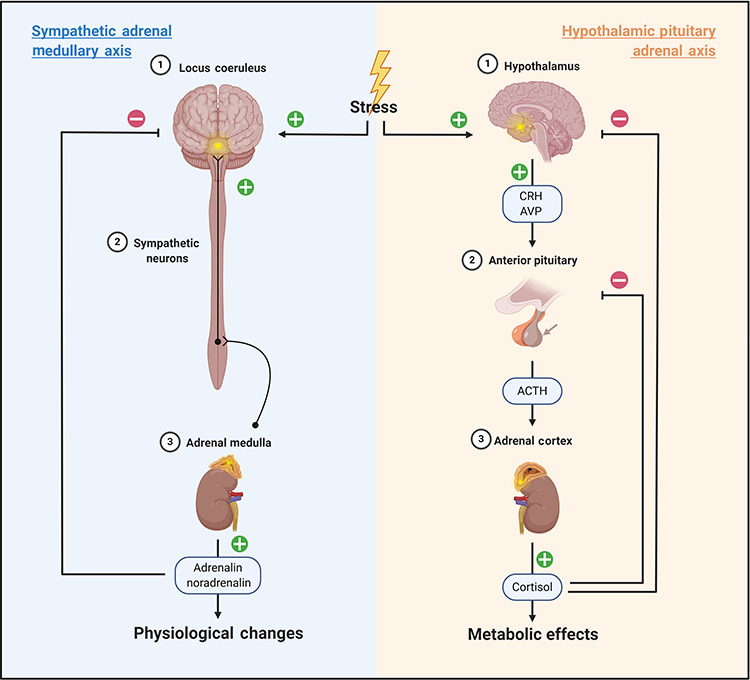
The sympathetic adrenal medullary axis and the hypothalamic pituitary adrenal axis are both altered following burn injury in children. ‘+’ indicates stimulatory pathways; ‘–’ indicates inhibitory pathways. Image created with BioRender.com. *ACTH* adrenocorticotrophic hormone, *CRH* corticotrophin-releasing hormone, *AVP* arginine vasopressin

The stress response can be divided into two pathways: the sympathetic-adrenomedullary (SAM) axis and the hypothalamic–pituitary–adrenal (HPA) axis [132] (Figure 2). Different mechanisms of action are utilized by each axis and markers involved in both pathways have been investigated in paediatric burns (Tables 4 and 5).

**Table 4:** Summary of reported abundances for biomarkers involved in the sympathetic adrenal medullary axis in children with burns

**Reference**	**Source**	**Reported normal limits**	**Abundance in children with burns**	**Age range**	**Time frame**	**Population TBSA (%)**
**Adrenaline**						
Fleming *et al.* (1992) [95]	Blood	<50 pg/mL	Elevated 147 pg/mL ± 36	11.1 years ±1.4	2 to 3 weeks post-burn	67 ± 6^a^
Gottschlich *et al.* (2002) [103]	Blood	10–200 pg/mL	Within normal limits 81–182 pg/mL	Children >3 years	For up to 4 weeks post-burn	53.2 ± 3.4^a^
Sedowofia *et al.* (1998) [22]	Blood	0.3–0.8 nmol/l	Elevated 1.3–6.4 nmol/l	5 months-12 years 5 months	Up to 108 hours after admission	20.5 ± 2.7^a^
Jeschke *et al.* (2012a) [45]	Urine (in 24 hours)	10 μg/day^†^	Elevated 25–115 μg/day^†^	0–18 years	Between 11 and 250 days post-burn	64 ± 12^b^
Gauglitz *et al.* (2009) [43]	Urine (in 24 hours)	10 μg/day^†^	Elevated 50–70 μg/day^†^	0–18 years	At least 2 months post-burn	57.9 ± 14.7^b^
Kulp *et al.* (2010) [24]	Urine (in 24 hours)	10 μg/day^†^	Elevated 38–65 μg/day^†^	8 years ±5	Up to 60 days post-burn	59 ± 17^a^
Norbury *et al.* (2008) [23]	Urine (in 24 hours)	8 μg/24 h^†^	Elevated 12–25 μg/day^†^	9.5 ± 5.1 (males) 6.7 ± 4.8 (females)	Up to 100 days post-burn	58.7 ± 16.9 (males)^a^ 56.8 ± 14.9 (females)^a^
Jeschke *et al.* (2011) [38]	Urine	10 μg/day^†^	Elevated 38–42 μg/day^†^	7.5 ± 5.3 years	Up to 60 days post-burn	50 ± 20^b^
**Noradrenaline**						
Gottschlich *et al.* (2002) [103]	Blood	80–520 pg/mL	Elevated 763–914 pg/mL	Children >3 years	Up to 2 weeks post-burn	53.2 ± 3.4^a^
Sedowofia *et al.* (1998) [22]	Blood	Not Reported	Elevated 2.3 nmol/l	5 months-12 years 5 months	Up to 6 hours after admission	20.5 ± 2.7^a^
Fleming *et al.* (1992) [95]	Blood	110–410 pg/mL	Elevated 867 pg/mL ± 113	11.1 years ±1.4	2 to 3 weeks post-burn	67 ± 6^a^
Gauglitz *et al.* (2009) [43]	Urine (in 24 hours)	40 μg/day^†^	Elevated 110–170 μg/day^†^	0–18 years	At least 2 months post-burn	57.9 ± 14.7^b^
Jeschke *et al.* (2012b) [134]	Urine	10 μg/day^†^	Elevated 50–150 μg/day^†^	9 ± 1 years	Up to 60 days post-burn	57 ± 3^a^
Kulp *et al.* (2010) [24]	Urine (in 24 hours)	15 μg/day^†^	Elevated 20–170 μg/day^†^	8 years ±5	Up to 2 years post-burn	59 ± 17^a^
Norbury *et al.* (2008) [23]	Urine (in 24 hours)	28 μg/24 h^†^	Elevated 81–110 μg/day^†^	9.5 ± 5.1 (males) 6.7 ± 4.8 (females)	Up to 100 days post-burn	58.7 ± 16.9 (males)^a^ 56.8 ± 14.9 (females)^a^
Jeschke *et al.* (2011) [38]	Urine	10 μg/day^†^	Elevated 25–105 μg/day^†^	7.5 ± 5.3 years	Up to 540 days post-burn	50 ± 20^b^
**Dopamine**						
Sedowofia *et al.* (1998) [22]	Blood	Not reported	Elevated 2.4 nmol/l	5 months-12 years 5 months	At 60 hours post-admission	20.5 ± 2.7^a^
Gottschlich *et al.* (2002) [103]	Blood	0–20 pg/mL	Elevated 371–4145 pg/mL	Children >3 years	Up to 4 weeks post-burn	53.2 ± 3.4^a^
Kulp *et al.* (2010) [24]	Urine (in 24 hours)	375 μg/day^†^	Reduced 150–205 μg/day^†^	8 years ±5	Up to 90 days post-burn	59 ± 17^a^

†Data derived from graph, ^a^Data presented as mean ± SEM, ^b^Data presented as mean ± SD

*TBSA* total body surface area

**Table 5:** Summary of reported abundances for biomarkers involved in the hypothalamic pituitary adrenal axis in children with burns

**Reference**	**Source**	**Reported normal limits**	**Abundance in burns**	**Age range**	**Time frame**	**Population TBSA (%)**
**AVP**						
Palmieri *et al.* (2006) [115]	Blood	<2 pg/mL	Within normal limits 2.2 ± 0.9 pg/mL	0–17 years	Admission to 8 weeks post-burn	41.8 ± 3.8^a^
Sedowofia *et al.* (1998) [22]	Blood	Not reported	Elevated 7.1–18.3 pmol/L	5 months-13 years	Admission to 18 hours, post-admission	20.5 ± 2.7^a^
**ACTH**						
Palmieri *et al.* (2006) [115]	Blood	3–50 ng/dL	Within normal limits 15.1 ± 6.9 ng/dL	0–17 years	Admission to 8 weeks post-burn	41.8 ± 3.8^a^
**Cortisol**						
Palmieri *et al.* (2006) [115]	Blood	5–20 μg/dl	Within normal limits 14.1 ± 4.6 μg/dl	0–17 years	2 months post-burn	41.8 ± 3.8^a^
Sedowofia *et al.* (1998) [22]	Blood	Not reported	Elevated 221.6–650.6 nmol/L	5 months-13 years	For up to 24 hours post-burn	20.5 ± 2.7^a^
Fleming *et al.* (1992) [95]	Blood	7–27 milligram/dL	Within normal limits 21.3 ± 1.6 milligram/dL	Mean age of 11.1 years	At mean of 12.6 days post-burn	67 ± 6^a^
Gottschlich *et al.* (2002) [103]	Blood	4–28 μg/dL	Elevated 24.1 ± 2 μg/mL	Children >3 years	Up to 4 weeks post-burn	53.2 ± 3.4^a^
Jeschke *et al.* (2008a) [37]	Blood	17.5 ng/mL^†^	Elevated 20–24.5 ng/mL^†^	8.0 ± 0.2 years	Up to 22 days post-burn	56 ± 0.3^a^
Jeschke *et al.* (2011) [38]	Blood	10 g/dL^†^	Elevated 20–43 g/dL^†^	7.5 ± 5.3 years	Up to 1100 days post-burn	50 ± 20^b^
Jeschke *et al.* (2008a) [37]	Urine	90 ng/mL^†^	Elevated 170–350 ng/mL^†^	8.0 ± 0.2 years	Up to 60 days post-burn	56 ± 0.3^a^
Jeschke *et al.* (2012a) [45]	Urine (in 24 hours)	5–21 μg/24 hours	Elevated 163 ± 56 μg/24 hours	0–18 years	Until 250 days post-burn	64 ± 12^b^
Jeschke *et al.* (2008b) [48]	Urine (in 24 hours)	Not reported	Elevated 185–430 μg/day^†^	0–18 years	Immediately after burn	>40^c^
Klein *et al.* (1995) [41]	Urine (in 24 hours)	8–47 mg/24 hours	Elevated 395 ± 284 mg/24 hours	5.8–17.5 years	3 weeks post-burn	63 ± 16^a^
Gauglitz *et al.* (2009) [43]	Urine (in 24 hours)	38 μg/day^†^	Elevated 139 ± 11 μg/24 hours	0–18 years	3 years post-burn	57.9 ± 14.7^b^
Jeschke *et al.* (2012b) [134]	Urine (in 24 hours)	Not reported	Elevated 80–300 μg/day^†^	Mean age 9 years	60 days post-burn	64 ± 12^a^
Norbury *et al.* (2008) [23]	Urine (in 24 hours)	10–70 μg/24 hours^†^	Elevated 145–284 μg/day^†^	9.5 ± 5.1 (males) 6.7 ± 4.8 (females)	Up to 100 days post-burn	58.7 ± 16.9 (males)^a^ 56.8 ± 14.9 (females)^a^
Jeschke *et al.* (2011) [38]	Urine	25 μg/day^†^	Elevated 75–175 μg/day^†^	7.5 ± 5.3 years	Up to 1100 days post-burn	50 ± 20^b^
Klein *et al.* (2004)	Urine	Maximum 50 μg/day	Elevated 371 ± 147 μg/day	7–18 years	Not recorded	>40^c^
**DHEA-S**						
Palmieri *et al.* (2006) [115]	Blood	10–140 μg/dL	Within normal limits 102.8 + _32.3 μg/dL	0–17 years	From admission to 8 weeks post-burn	41.8 ± 3.8^a^

†Data derived from graph, ^a^Data presented as mean ± SEM, ^b^Data presented as mean ± SD, ^c^Data presented as minimum value

*TBSA* total body surface area, *AVP* arginine vasopressin, *DHEA-S* dehydroepiandrosterone sulphate

#### Sympathetic-adrenomedullary axis

SAM axis activation utilizes neural circuitry and catecholamines to rapidly affect physiology [133]. Sympathetic innervation of the adrenal medulla stimulates synthesis and release of catecholamines into the blood, where they can be transported throughout the body to elicit the stress response [133].

##### Catecholamines (Adrenaline, Noradrenaline and Dopamine)

Catecholamines are hormonal neurotransmitters produced in the adrenal medulla that play a major role in the SAM axis of the stress response. Specific catecholamines, such as adrenaline (epinephrine), noradrenaline (NA; norepinephrine) and dopamine, have been used within medical research as indicators of stress and, in general, increased concentrations of total catecholamines have been observed in children with burns [95, 134] (Table 4).

Adrenaline represents 80% of the catecholamines secreted by the adrenal medulla in humans [133]. Several studies have reported that adrenaline increased after a burn injury in the paediatric population [22, 24, 38]; however, one study performed by Gottschlich et al. (2002) reported serum adrenaline levels to be within normal limits [103]. Urinary levels of adrenaline have been reported to increase up to 10-fold following a burn [43], and remain elevated up to 250 days post-burn [45], whereas serum adrenaline levels have been reported to stay elevated up to 3 weeks post-burn [95].

NA has generally been observed to increase in children with burns [38, 95, 103]; however, there is conflicting evidence regarding how long NA remains elevated after a burn. Sedowofia et al. (1998) reported elevated levels of NA in blood at admission that returned to normal levels after 6 hours [22]. Conversely, in a study performed by Kulp et al. (2010), urinary NA was elevated in children for up to 2 years after discharge from the hospital [24]. Urinary NA has been reported to increase up to 4-fold following a burn, returning to normal between 2 and 6 months post-burn [43].

Dopamine, the precursor to NA, has been evaluated in children with burns; however, the results are conflicting. One study reported that urinary dopamine was significantly reduced for up to 90 days post-burn in children [24], whereas another study found that serum dopamine was elevated for the first 4 weeks post-burn [103]. Discrepancies may be due to the sampling method as Kulp et al. (2010) evaluated total urinary dopamine in 24 hours [24], whereas Gottschlich et al. (2002) evaluated a single time point blood sample [103]. In a study of the first 108 hours post-burn, dopamine levels were observed to fluctuate between 0.05 nmol/L and 18.8 nmol/L [22]. Consistent sampling techniques should be used to elucidate accurate information regarding dopamine response after a burn in children. Levels of both NA and dopamine have been positively correlated with burn size in children aged up to 11 years 2 months [135]. Interestingly, no such relationship was observed between adrenaline and TBSA. Some studies have reported that catecholamines are higher in males than in females after a burn [68]. However, discordant results have been reported for dopamine and adrenaline, with no statistically significant difference detected in these markers between males and females with burns [68]. It would be beneficial to identify whether other characteristics influence catecholamine response to burns such as age, burn depth or burn mechanism.

Although these are commonly used markers of stress in the paediatric burn population, there is little consensus on what is considered a normal range for these markers. This makes it difficult to consolidate information from different studies and evaluate their diagnostic or prognostic utility. Additionally, these markers are influenced by numerous environmental cues and stressors, meaning that they are not specific to burn-related stress and it may be difficult to use them diagnostically for stress in burns. Conversely, it may not be wise to discount these markers, as any stress being experienced by a child should be treated, irrespective of the cause.

##### Salivary Alpha-amylase

Alpha-amylase is a salivary enzyme that has been used previously to evaluate stress, as it is an indirect marker of SAM axis activation [136]. In paediatric burns, only one study has investigated salivary alpha-amylase (sAA). Brown et al. (2012) evaluated sAA levels in paediatric outpatients with burns <15% TBSA. It was observed that over the course of a single dressing change, sAA increased. Furthermore, when sAA levels were evaluated over the course of three dressing changes as healing progressed, sAA was observed to elevate at each subsequent dressing change [7]. This suggests that anticipatory stress may increase over the course of treatment. Alpha-amylase could have great potential for analysing stress in children, as saliva is a non-invasive biological tissue to collect [137, 138]; however, additional research is required to determine its efficacy in identifying stress in a paediatric burn population.

##### Hypothalamic–pituitary–adrenal Axis

The HPA axis is the secondary molecular pathway responsible for the stress response that provides long-lasting physiological changes [133] (Figure 2). The hypothalamus produces oxytocin, corticotrophin-releasing hormone (CRH) and arginine vasopressin (AVP) which signal the anterior pituitary gland to secrete adrenocorticotrophic hormone (ACTH) into the blood. This stimulates the adrenal cortex to produce cortisol, which acts on various tissues in the body, causing the physiological changes associated with the stress response, such as increased heart rate [139].

In the context of paediatric burns, CRH, AVP and ACTH have not been extensively investigated. One study, performed by Sedowofia et al. (1998) observed an increase in AVP in the blood of children with burns at the time of admission that remained elevated for up to 18 hours post-burn, before returning to normal [22]. Furthermore, Smith et al. (1997) observed that the serum level of AVP at admission was positively correlated with the size of the burn [135], whereas another study reported that serum AVP remained within normal limits in the 8 weeks following >20% TBSA burns in children [115]. In the same study, ACTH was also reported to remain within normal limits for the extent of the study period. To our knowledge, CRH levels have not been evaluated in a paediatric population after burn injury.

##### Cortisol

Cortisol is the end-product of the HPA axis and is therefore the most discussed and widely accepted marker for the detection and evaluation of stress. Within the paediatric burns population, cortisol has most commonly been evaluated in blood [115] and urine [23]; however, it has also been detected in saliva [7] (Table 5). In children with burns, urinary cortisol levels have consistently been observed to increase following burn injury [41, 48, 74]. In some studies, urinary cortisol has been observed to remain elevated for months [45, 134] and even years [38, 43] after the burn occurred. Similarly, blood cortisol levels have been observed to either increase [22, 103] or remain within normal limits [95, 115]. Contrary to this, a study that evaluated cortisol levels in the saliva of children with burns observed an acute decrease in cortisol levels after dressing changes [7]. In that study, saliva samples were collected during morning outpatient burn clinics, when cortisol levels naturally decrease. Sampling time is a crucially important factor when measuring cortisol as cortisol secretion is subject to circadian influence. Cortisol levels fluctuate throughout the day [140, 141] generally peaking 40–45 minutes after waking and then steadily decreasing throughout the day, in a process known as the cortisol awakening response (CAR) [141].

Aside from sampling time, other confounding variables need to be evaluated. Jeschke et al. (2008b) have shown that age can impact cortisol levels [48]. Adolescents (aged 10–18 years) were observed to have significantly higher levels of 24-hour urine cortisol up to 60 days post-burn, compared with toddlers (aged 0–3.9 years) and prepubertal children (aged 4–9.9 years). Additionally, gender has been observed to influence cortisol secretion in children with burns greater than 40% TBSA, with females displaying significantly lower levels of 24-hour urinary cortisol for up to 200 days post-burn [68]. Similarly, Norbury et al. (2008) reported higher urinary cortisol levels in males following burn injury [23]. Interestingly, an earlier study performed in the same laboratory observed no gender-specific differences in blood cortisol levels at discharge and 6, 9, 12, 18 and 24 month follow-ups in a similar cohort [109]. This suggests that the source of cortisol (e.g. blood or urine) is an important experimental condition that needs to be considered. Finally, an unfamiliar setting such as a hospital may also influence cortisol levels and should be considered.

Dehydroepiandrosterone (DHEA) and dehydroepiandrosterone sulphate (DHEA-S) are steroids produced by the adrenal glands in response to ACTH stimulation, like cortisol. Only one study has evaluated the response of DHEA/-S to burn injury in a paediatric population, and those authors reported that admission levels of DHEA-S were within normal limits [115].

### Limitations and future research

Many potential biomarkers have been identified that change in response to burn injury in children, however, further research is needed to comprehensively understand the underlying biology of paediatric burns, identify markers suitable for clinical use and translate these findings into diagnostic or prognostic tools to implement for rapid patient management. Currently, there are several caveats within the literature that are limiting biomarker translational progress.

Much of the research aims to determine the longitudinal changes in biology following burn injury. While some studies focus more on acute phase healing, others are interested in long-term changes. Biological responses during both phases are important; however, it can be difficult to consolidate the findings of these studies. Acute phase healing studies have narrower time points (often daily), whereas long-term studies have much wider time points (up to 1 month). When trying to identify changes in biological response, having such temporally distinct time points can make it difficult to compare studies, as some studies are less sensitive to the acute changes.

Some biomarkers, such as GH or cortisol, are regulated by circadian rhythm and therefore have a distinct pattern of fluctuation. This fluctuation can create inaccuracies when utilizing dynamic samples such as blood, urine or saliva as they only provide transient information on concentrations. Difficulties can arise when trying to analyse these markers if there are variations in the times that samples were collected. As such, additional care is needed when evaluating these markers, and the time of sample collection needs to be clearly defined. As a result, baseline concentration of these markers is difficult to assess, and often multiple samples need to be collected over the course of the day [142]. For example, as cortisol peaks between 30–45 minutes after waking, the peak cortisol production can be measured by taking multiple samples within the first hour after waking.

Several studies report transient elevations of stress markers in children who have suffered a burn injury; however, there is a lack of psychological assessments used in paediatric burns literature to evaluate whether stress experienced by children with burns is directly related to the biomarkers identified. Consequently, it remains unclear how these markers correlate to psychological impairment in paediatric burn patients. As fluctuations in stress marker production does not always lead to psychological disorders, it is important to understand why some children develop psychological issues following a burn, while other children do not. Future research should focus on psychological testing alongside biomarker evaluation to determine how fluctuations of stress markers correlate with adverse psychological outcomes and to enable identification of children at risk. Additionally, genetic and epigenetic markers could explain the fluctuations in stress markers observed between children, and why only some children will develop PTSD. Future biomarker research should incorporate studies of patient DNA to explore these mechanisms.

Issues relating to study design impact upon the ability to compare previous research. First, there is significant variation in reported normal/control ranges for most of the markers, as studies source their ‘normal’ levels from different populations. Some studies use normal values obtained from the hospital where the study took place, while other studies have a control cohort that they test alongside their patient cohort. It is assumed that the control cohort is a reflection of the patient cohort, minus the ailment being studied, meaning that certain characteristics should not significantly differ between the two cohorts (such as age, gender, ethnicity, etc.). However, this is not always the case and can distort the results of the study if not accounted for. Within the literature reviewed here, the control cohort often includes children undergoing non-burn-related surgery (such as elective surgeries for orthopaedic corrections [41], plastic surgery [21] or inguinal hernia repair [82]). Other studies omit the normal values that they used [40]. Additionally, studies reporting similar normal values can differ by up to three orders of magnitude [36, 37]. This issue is not specific to paediatric burns research, as even studies that are primarily designed to assess values of specific markers in healthy children, particularly cytokines, are discordant [143, 144].

Many studies report biological changes that occur in severe paediatric burns. Severity can be measured using several different characteristics (such as whether surgery or grafting is required, or if the patient requires admission); however, the information provided in each of the studies varies, making it difficult to classify severity in the same way across all studies. One measure of severity that is often presented in all studies is the size of the burn. Unfortunately, the threshold whereby burns are considered severe is not consistent within the literature. Most commonly, >40% surface area is considered severe; however, Gottschlich et al. (2002) included patients with TBSA as low as 25% [103]. Conversely, Gore et al. (2001) considered burns of >60% TBSA as severe [145]. Only one study specified burn depth in conjunction with TBSA as an inclusion criteria for their study on severe burns [36]. A standardized definition of ‘severe’ (including different categorical classifications of burns that directly reflect the biology, i.e. TBSA, depth, etc.) is required, otherwise comparability of the studies will become unnecessarily complex.

The primary biological tissue used to evaluate biomarkers in paediatric burns is blood. For hospitalized patients, blood may be a valuable source of biomarkers; however, the invasive nature of blood collection prevents it from being a useful prognostic medium for patients who are being treated without needing to be cannulated or undergo surgery. In an outpatient setting, where most paediatric burn injuries are treated [146], it is difficult to collect blood from children and would therefore render any blood test unusable. In terms of the psychological impact, it is known that blood tests cause distress in children [147]. This poses the question, is blood the best medium for diagnostic tests in children? It is somewhat surprising that more studies have not focused on using more child-friendly biosamples. There is substantial research that uses children’s urine as a diagnostic sample; however, this is most commonly only to measure stress-related markers. Only one study, that of Kulp et al. (2010), evaluated urine inflammatory cytokines [24]. In addition to investigating blood-based biomarkers for paediatric burn treatment, future studies should also focus on expanding the use of additional non-invasive biosamples such as urine and saliva [137, 138, 148]. In doing so, our understanding of the expression and abundance of the already identified markers would be improved. This will undoubtedly require substantial research as markers identified in blood may have different abundance profiles in other bodily fluids [149].

Various methods were used to measure different markers, including ELISA [80], high-performance liquid chromatography–tandem mass spectrometry [7], nephelometry [40, 150], radioimmunoassay [41, 95, 150], high-performance liquid chromatography [22, 41, 43], and surface plasmon resonance imaging [76]. Many of the studies that quantified cytokines utilized the Bio-Rad Bio-Plex Suspension Assay and this resulted in similar results for these cytokines across studies. In comparison, other studies that utilized methods such as ELISA, detected cytokines at a much lower concentration.

Another factor that limits the comparability of studies is that many do not provide adequate information about their study population including the ethnicity or gender of the cohort, mechanism of burn, or the burn depth. All these factors could potentially influence the biological response, some of which have already been documented [65]. In moving forward, consistent reporting of normal values, burn classifications, sampling techniques and analysis methods need to be used to elucidate useful information regarding biomarker response after a burn injury in children.

Much of the research reported in the paediatric burns literature consists of targeted or directed quantification of biomarkers, where specific biomarkers are prospectively targeted for study or measurement. Although this is important for understanding their individual response to burn injury, additional discovery-type studies should be performed to identify other potential biomarkers influenced by burn injury that may not be as intuitive. In doing so, the underlying biological implications of burn injury could be more comprehensively evaluated. Furthermore, studies already performed in adults should be replicated in children to identify the similarities in response to burn injury.

While many biomarkers have been investigated in children’s burns, knowledge of the synergistic and antagonistic interactions of the identified biomarkers is incomplete. Understanding biomarker interactions is necessary to develop meaningful diagnostic and prognostic tests. Some markers discussed in this review significantly alter or control the expression of other markers, which makes it difficult to single out individual markers for clinical use. However, this could be rectified by utilizing panels of biomarkers for clinical analysis rather than individual biomarkers. This potentially allows for the development of a more robust method of evaluating burn injury. Obviously, this requires far more research to identify and validate any biomarker panels that may be of diagnostic or prognostic use.

## Conclusions

Research conducted within the paediatric burn space has the potential to make a significant impact on the lives of children affected by burn injuries. Although there is a large amount of research surrounding the biological response to burns, additional research is still required to translate this knowledge into clinically relevant diagnostic tests. It is important that in the future, research is conducted in a way that will allow for comparisons to be made between studies, to create a thorough understanding of the biological response to burn injury in paediatric patients. Only when we have this understanding will clinical translation be possible. Through understanding these healing processes and identifying such biomarkers, burn treatment could be improved to provide more personalized care and better management of stress and pain during treatment.

## Ethics approval and consent to participate

Not applicable.

## Consent for publication

Not applicable.

## Availability of data and materials

Not applicable.

## Competing interests

The authors declare that they have no competing interests.
